# Differential effects of the LncRNA RNF157-AS1 on epithelial ovarian cancer cells through suppression of DIRAS3- and ULK1-mediated autophagy

**DOI:** 10.1038/s41419-023-05668-5

**Published:** 2023-02-20

**Authors:** Pengfei Xu, Sujuan Xu, Haiyue Pan, Chencheng Dai, Yiran Xu, Luyao Wang, Yu Cong, Huilin Zhang, Jian Cao, Lili Ge, Xuemei Jia

**Affiliations:** 1 Nanjing Maternity and Child Health Care Institute, Women’s Hospital of Nanjing Medical University (Nanjing Maternity and Child Health Care Hospital), 210004 Nanjing, China; 2 Department of Clinical Laboratory, Women’s Hospital of Nanjing Medical University (Nanjing Maternity and Child Health Care Hospital), 210004 Nanjing, China; 3 Department of Gynecology, Women’s Hospital of Nanjing Medical University (Nanjing Maternity and Child Health Care Hospital), 210004 Nanjing, China

**Keywords:** Autophagy, Ovarian cancer

## Abstract

Analyses of several databases showed that the lncRNA RNF157 Antisense RNA 1 (RNF157-AS1) is overexpressed in epithelial ovarian cancer (EOC) tissues. In our study, suppressing RNF157-AS1 strikingly reduced the proliferation, invasion, and migration of EOC cells compared with control cells, while overexpressing RNF157-AS1 greatly increased these effects. By RNA pulldown assays, RNA binding protein immunoprecipitation (RIP) assays, and mass spectrometry, RNF157-AS1 was further found to be able to bind to the HMGA1 and EZH2 proteins. Chromatin immunoprecipitation (ChIP) assays showed that RNF157-AS1 and HMGA1 bound to the ULK1 promoter and prevented the expression of ULK1. Additionally, RNF157-AS1 interacted with EZH2 to bind to the DIRAS3 promoter and diminish DIRAS3 expression. ULK1 and DIRAS3 were found to be essential for autophagy. Combination autophagy inhibitor and RNF157-AS1 overexpression or knockdown, a change in the LC3 II/I ratio was found using immunofluorescence (IF) staining and western blot (WB) analysis. The autophagy level also was confirmed by autophagy/cytotoxicity dual staining. However, the majority of advanced EOC patients require platinum-based chemotherapy, since autophagy is a cellular catabolic response to cell stress. As a result, RNF157-AS1 increased EOC cell sensitivity to chemotherapy and death under cis-platinum (DDP) treatment by suppressing autophagy, as confirmed by cell count Kit-8 (CCK8) assays, flow cytometry, and autophagy/cytotoxicity dual staining. Therefore, the OS and PPS times were longer in EOC patients with elevated RNF157-AS1 expression. RNF157-AS1-mediated autophagy has potential clinical significance in DDP chemotherapy for EOC patients.

## Introduction

Epithelial ovarian cancer (EOC) is the most lethal gynecological cancer [1]. Annually worldwide, 230,000 women are diagnosed with EOC, and 150,000 die [2]. One of the main factors contributing to the high death-to-incidence rate is the advanced stage at diagnosis (~75% of EOC patients). Late-stage EOC has a 5-year survival rate of 29%, in contrast with 92% for early-stage EOC. Despite advances in medical care and research in EOC, the 5-year survival rate has increased from 30% to 46% [3]. EOC is one of three cancers in women that has an increasing trend in mortality in China (breast cancer, cervical cancer, and EOC) [3]. Therefore, it is still necessary to further study the molecular mechanism of EOC.

A maternally imprinted gene, DIRAS family GTPase 3 (DIRAS3, also named Aplasia Ras homolog member I, ARHI), is involved in several steps in autophagy. More than 60% of ovarian cancers exhibit downregulated DIRAS3. Lu Z. et al. discovered that DIRAS3 blocks PI3K, inhibits mTOR activation, and increases ATG4 expression to regulate autophagy in EOC cells and induce tumor dormancy [4]. DIRAS3 also forms an autophagosome initiation complex with PIK3C3, BECN1, and ATG14 in dormant EOC cells [5]. In addition, DIRAS3 can induce autophagy by downregulating the epidermal growth factor receptor, inhibiting PI3K and Ras/MAP signaling, and activating FOXo3a-mediated Rab7 expression [6]. Therefore, DIRAS3 plays a pivotal role in the autophagy of EOC cells. In addition to autophagy, DIRAS3 is also associated with migration. D.B. Badgwell and colleagues reported that DIRAS3 suppresses the migration of ovarian cancer cells via the STAT3 and FAK/Rho signaling pathways [7]. M. Klingauf et al. suggested that DIRAS3 can interact with C-RAF and downregulate MEK to suppress cell migration [8]. Additionally, overexpression of DIRAS3 inhibits the expression of β-1 integrin, which is associated with migration, adhesion, and invasion [9].

The earliest autophagy-specific complex consists of unc-51-like kinase 1 (ULK1), autophagy-related protein 13 (ATG13), focal adhesion kinase family interacting protein of 200 kDa (FIP200), and ATG10. Upon autophagy induction, the ULK1 complex translocates for autophagy initiation and regulates the autophagy process. In ovarian cancer cells, inhibiting autophagy regulator—ULK1, can decrease the autophagy, and repress viability and suspension growth of high-grade serous ovarian cancer (HGSOC) cells [10, 11]. Chemoresistance in ovarian cancer may be enhanced by ULK1-mediated autophagy [12].

Autophagy is a lysosomal degradation pathway through which damaged or superfluous cell components are degraded into basic biomolecules, which are then recycled into the cytosol [13]. Autophagy enhances cell survival under unfavorable conditions. For instance, autophagy increases cell survival when cells are deprived of serum, amino acids, or growth factors [14]. Autophagy is increased in EOC cells under DDP exposure to increase overall survival [15, 16]. Autophagy, however, has been shown to promote apoptosis and death in cancer cells [17, 18]. Furthermore, autophagy promotes EOC cell dormancy [19]. Dormant cells exhibit decreased proliferation, while they can still exhibit enhanced survival under harsh environmental conditions. Thus, whether autophagy increases or decreases cell survival depends on the nutritional status of the cell, the type of cell, the stage or environment in which the tumor is developing, and changes in the expression of oncogenes or suppressor genes [20, 21].

The lncRNA RNF157 Antisense RNA 1(RNF157-AS1) was found to be highly expressed in EOC tissues in our previous studies [22]. In addition, RNF157-AS1 was overexpressed in GSE_14407, GSE_74448, and the TCGA database compared with the GTEx database. In our study, RNF157-AS1 knockdown was found to inhibit the proliferation, invasion, and migration of SKOV3 and A2780 cells, while RNF157-AS1 overexpression promoted the proliferation, invasion, and migration of EOC cells. However, RNF157-AS1 had no effect on the apoptosis of EOC cells. Further studies demonstrated that RNF157-AS1 can bind to the EZH2 and HMGA1 proteins and suppress DIRAS3 and ULK1 expression. ULK1 and DIRAS3, as autophagy initiation regulators, play pivotal roles in autophagy. However, under treatment with cis-platinum (DDP), RNF157-AS1 instead promoted EOC cell chemosensitivity and apoptosis after inhibiting autophagy. Taken together, these findings indicated that RNF157-AS1 induced EOC cell proliferation, invasion, and migration by blocking autophagy, which could contribute to DDP resistance in EOC. This study establishes the role of the lncRNA RNF157-AS1 in the regulation of cell vitality and autophagy under differential conditions and provides insights into chemoresistance and autophagy in EOC.

## Results

### RNF157-AS1 is overexpressed in EOC tissues

**Fig. 1:**
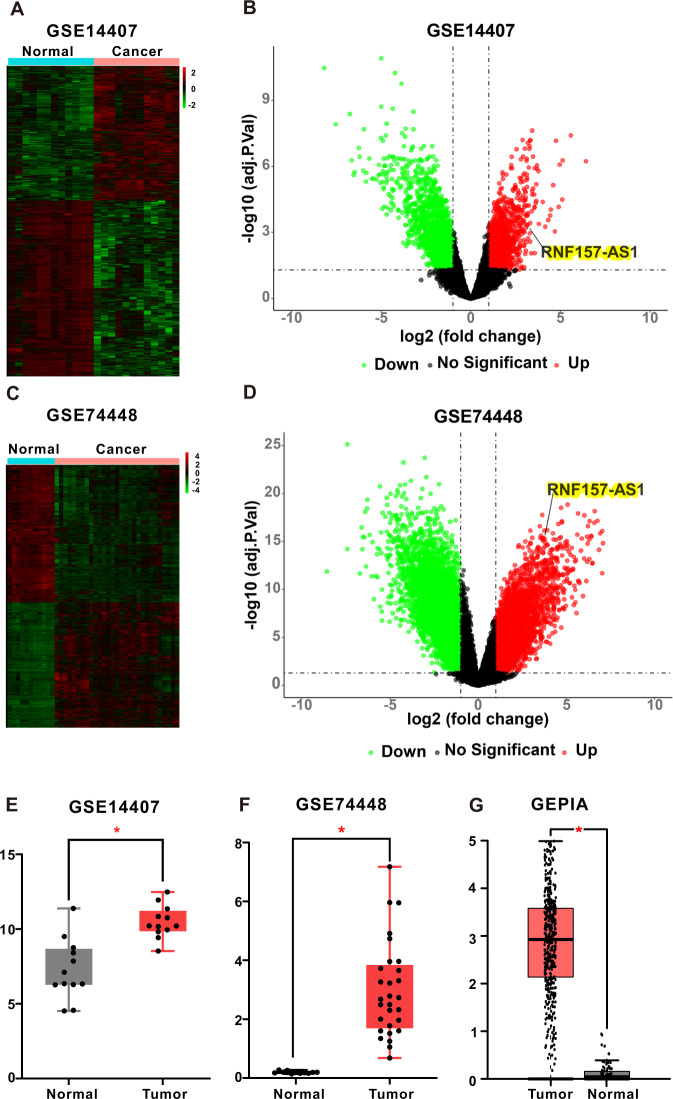
RNF157-AS1 was overexpressed in EOC tissues. **A** Differentially expressed lncRNAs in GSE_14407 with hierarchical clustering. **B** Differentially expressed lncRNAs in GSE_14407 shown on a volcano plot and including RNF157-AS1. **C** A heatmap was used to show the differentially expressed lncRNAs in GSE_74448. **D** A volcano plot was used to visualize the differentially expressed lncRNAs in GSE_74448, including RNF157-AS1. **E–G** RNF157-AS1 was overexpressed in 12 CEPI samples compared with 12 OSE samples in GSE_14407 (**E**), in 29 EOC tissues compared with normal ovarian tissues in GSE_74448 (**F**), and in 426 EOC tissues in the TCGA database compared with 88 normal ovarian tissues in GTEx database (**G**).

In our previous study, RNF157-AS1 was found to be overexpressed in EOC tissues compared with normal ovarian and benign ovarian cyst tissues [22]. In addition, RNF157-AS1 was also overexpressed in 12 laser-capture microdissected serous ovarian cancer epithelial samples (CEPI) samples compared with 12 ovarian surface epithelial (OSE) samples in GSE_14407 and increased in 29 ovarian cancer tissues compared with normal ovarian tissues in GSE_74448 (Fig. 1A–F). Moreover, via the gene expression profiling interactive analysis (GEPIA, http://gepia.cancer-pku.cn/) database, we found that RNF157-AS1 had higher levels in 426 serous ovarian cancer tissues in the TCGA database than 88 normal ovarian tissues in the GTEx database (Fig. 1G). Thus, these data indicated that the lncRNA RNF157-AS1 is overexpressed in EOC tissues. However, it is still unknown whether RNF157-AS1 has a function in cancer, especially in EOC.

### The effect of RNF157-AS1 on EOC cell proliferation, apoptosis, invasion, and migration

**Fig. 2:**
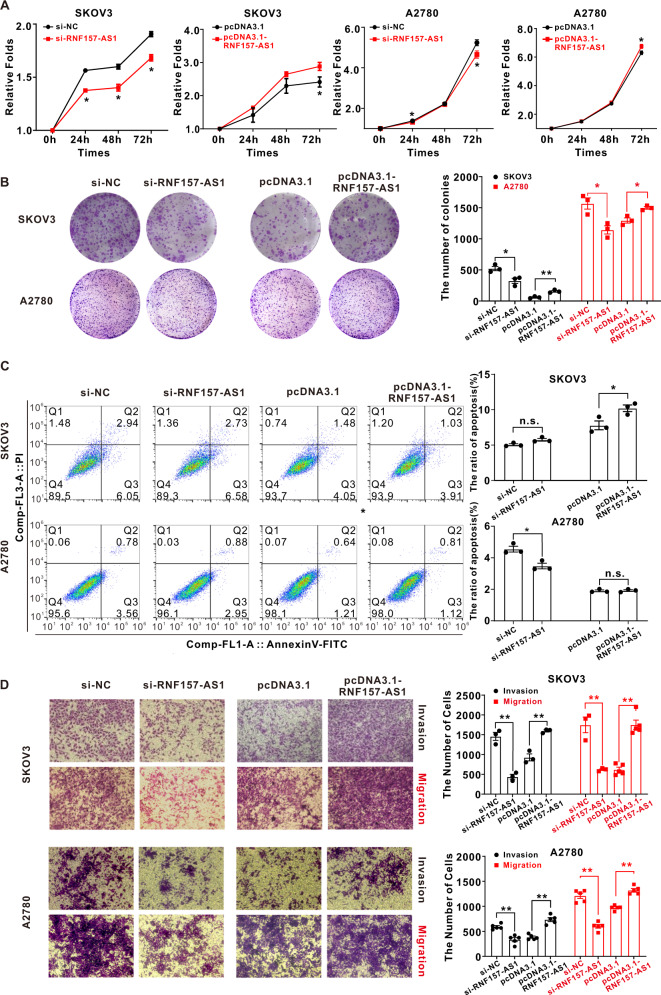
The effect of RNF157-AS1 on EOC cell proliferation, apoptosis, invasion, and migration. **A** The CCK8 assay showed that RNF157-AS1 inhibition led to a significant decrease in the viability of SKOV3 and A2780 cells while RNF157-AS1 overexpression increased the proliferation rate of EOC cells. **B** The clone formation assay indicated that RNF157-AS1 knockdown significantly decreased the viability of SKOV3 and A2780 cells while RNF157-AS1 overexpression enhanced the proliferation of SKOV3 and A2780 cells. **C** The flow cytometry assay suggested RNF157-AS1 knockdown or overexpression had little effect on EOC cell apoptosis. **D** The transwell assay showed that RNF157-AS1 overexpression enhanced the invasion and migration of SKOV3 and A2780 cells but RNF157-AS1 depletion attenuated the invasion and migration of SKOV3 and A2780 cells. Data were obtained from at least three independent experiments. *P < 0.05; **P < 0.01 (*t*-test).

To further determine the function of RNF157-AS1 in EOC cells, we first confirmed that RNF157-AS1 indeed has no protein-coding ability according to UCSC (Fig. S1). Then we used in vitro RNA interference (RNAi) or the RNF157-AS1 plasmid to silence or overexpress RNF157-AS1, respectively, by transfection of EOC cells (Table S1 and Fig. S2A–S2D). The cell counting Kit-8 (CCK8) results suggested that RNF157-AS1 depletion led to decreasing in EOC cell viability. Conversely, RNF157-AS1 overexpression enhanced the proliferation of EOC cells (Figs. 2A and S2E). These results were also confirmed by the clone formation assay (Figs. 2B and S2F). However, the flow cytometry results indicated that neither knockdown nor overexpression of RNF157-AS1 affected SKOV3 and A2780 cell apoptosis (Fig. 2C). Transwell was further utilized to determine the effects of RNF157-AS1 on the invasion and migration of EOC cells. The invasion and migration of SKOV3 and A2780 cells were reduced after RNF157-AS1 depletion, while overexpression of RNF157-AS1 enhanced the invasion and migration of EOC cells (Fig. 2D). This finding was further supported by the results of the wound-healing assay of SKOV3 and OVCAR3 cells (Fig. S2G).

### RNF157-AS1 interacts with HMGA1 and EZH2 in the nucleus

**Fig. 3:**
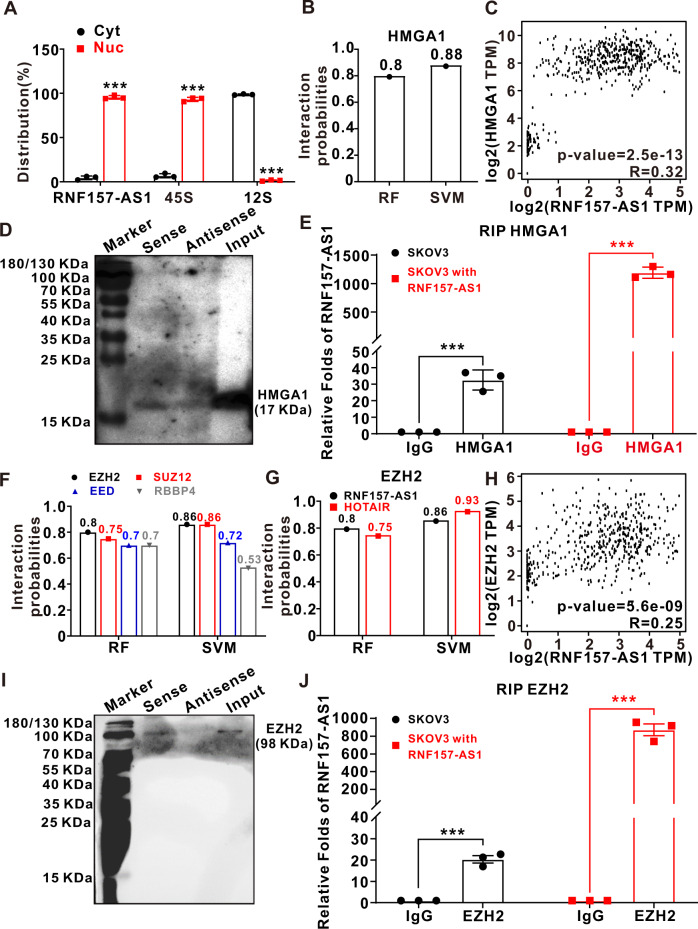
RNF157-AS1 interacted with HMGA1 and EZH2 in the nucleus. **A** Subcellular localization analysis suggested that RNF157-AS1 was localized mainly in the nucleus in EOC cells. **B** The RPISeq database showed that RNF157-AS1 had a very high interaction predicting score with HMGA1 protein (RF:0.8; SVM:0.88). **C** RNF157-AS1 expression had a positive correlation with HMGA1 expression (*R* = 0.32, *P* = 2.5*10^−13^) according to the GEPIA database. **D** Western blot analysis showed that RNF157-AS1 pulled down a greater amount of protein than did the control. **E** The RIP assay suggested HMGA1 interacted with RNF157-AS1 in not only RNF157-AS1 overexpression but also control EOC cells. **F** According to the RPISeq database, EZH2 had the highest interaction predicting score with RNF157-AS1 among the four subunits of the PRC2 complex. **G** EZH2 had a similar interaction predicting scores between RNF157-AS1 and HOTAIR; **H** RNF157-AS1 had a positive correlation with HMGA1 (*R* = 0.25, *P* = 5.6*10^−9^) according to the GEPIA database. **I** Western blot analysis showed that EZH2 was present only in the RNF157-AS1 (sense) precipitate compared with the control (antisense) precipitate. **J** The RIP assay suggested EZH2 interacted with RNF157-AS1 in not only RNF157-AS1 overexpressing but also control EOC cells. Data were obtained from at least three independent experiments. ***P < 0.001 (*t*-test).

To elucidate the mechanism of RNF157-AS1 in EOC cells, we first assessed the subcellular localization of RNF157-AS1. The real-time quantitative PCR (qPCR) results revealed that RNF157-AS1 is localized mainly in the nucleus in EOC cells (Fig. 3A). Since lncRNAs mainly play its roles through binding proteins, we performed an RNA pulldown assay to identify the proteins that RNF157-AS1 could bind directly. Relative to the control construct, 17 proteins were identified to directly bind to RNF157-AS1 (Table S2). Among them, high mobility group A1 (HMGA1) attracted our attention not only because it is localized in the nucleus but also because it had a very high predicted score according to the RNA–protein Interaction Prediction (RPISeq) website (http://pridb.gdcb.iastate.edu/RPISeq/) (Fig. 3B). Additionally, there was a positive correlation between HMGA1 and RNF157-AS1 expression in EOC tissues (Fig. 3C). We further used the western blot to measure the HMGA1 levels from the RNF157-AS1 (sense) and control precipitates (antisense). The results showed that RNF157-AS1 can interact with HMGA1 (Fig. 3D). The RNA binding protein immunoprecipitation (RIP) assay was further utilized to indicate the association of RNF157-AS1 with HMGA1. As shown in Fig. 3E, in both control and overexpression EOC cells, the RNF157-AS1 level was prominently increased by HMGA1 antibody precipitation. These results elucidated that RNF157-AS1 indeed interacted with HMGA1 directly.

A previous study reported that ~20% of all human lncRNAs can interact with the PRC2 complex [23] which indicates that lncRNAs have a general role in recruiting the PRC2 complex to their target genes. The PRC2 complex contains at least four proteins (EZH2, SUZ12, EED, and RBBP4). By using the RPISeq website, we first determined which PRC2 complex component was most likely to bind to RNF157-AS1. By prediction, EZH2 had the highest score (Fig. 3F). Moreover, the predictive algorithm score for EZH2 and RNF157-AS1 is equal to the score between EZH2 and HOTAIR (Fig. 3G), which has been reported to interact with EZH2 [24]. Moreover, there was a positive correlation between EZH2 and RNF157-AS1 expression in EOC tissues (Fig. 3H). Then as shown in Fig. 3I, we detected the EZH2 protein only in the RNF157-AS1 precipitate (sense group). In addition, the RIP assay revealed that the RNF157-AS1 expression level was ~16-fold higher in the EZH2 group than in the IgG group. Moreover, when RNF157-AS1 was overexpressed in SKOV3 cells, the RNF157-AS1 level was strikingly increased in the EZH2 antibody precipitate, approximately 1000-fold when compared to that in the IgG precipitate (Fig. 3J). Therefore, RNF157-AS1 could also combine with the EZH2 protein.

### RNF157-AS1 serves as a modular scaffold to epigenetically repress DIRAS3 and ULK1

**Fig. 4:**
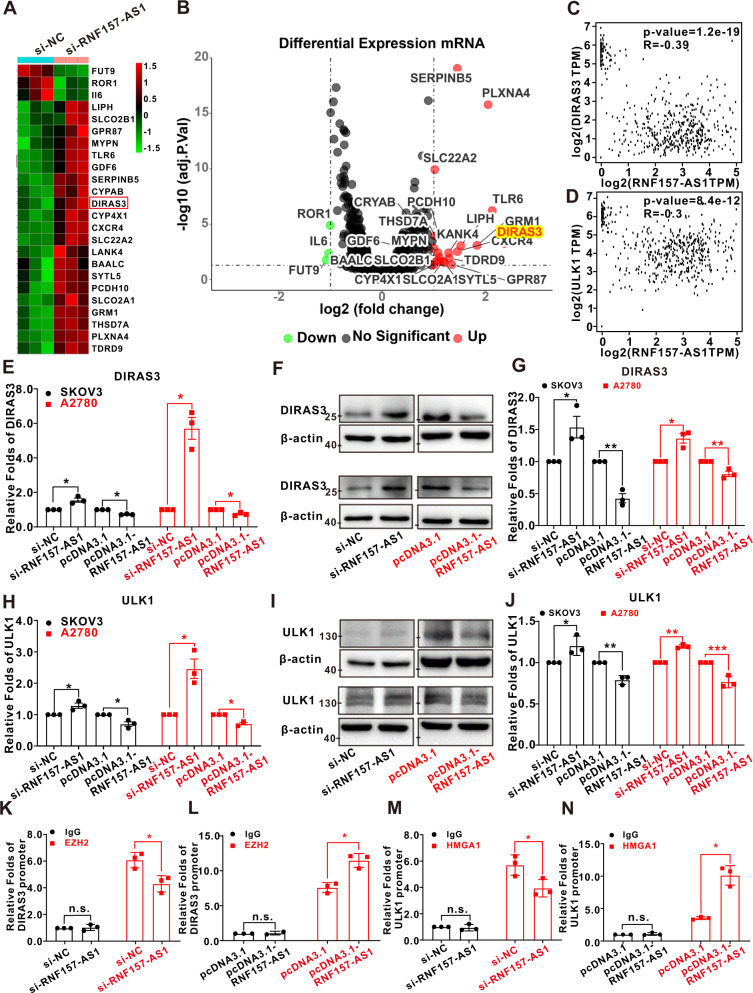
RNF157-AS1 served as a modular scaffold to epigenetically suppress DIRAS3 and ULK1 expression. **A** Differentially expressed mRNAs after RNF157-AS1 knockdown with hierarchical clustering. **B** Differentially expressed mRNAs shown on a volcano plot. **C** and **D** RNF157-AS1 expression had a negative correlation with DIRAS3 expression (*R* = −0.39, *P* = 1.2*10^−19^) and ULK1 expression (*R* = −0.3, *P* = 8.4*10^−12^). **E–G** The mRNA and protein expression level of DIRAS3 after RNF157-AS1 knockdown or overexpression. Full-length blots are presented in Supplementary Figs. S5 and S6. **H–J** The mRNA and protein expression level of ULK1 after RNF157-AS1 knockdown or overexpression. Full-length blots are presented in Supplementary Figs. S5 and S6. **K** and **L** ChIP-qPCR assay demonstrated that inhibiting RNF157-AS1 suppressed the binding of EZH2 to the promoter of the DIRAS3 genes and that RNF157-AS1 overexpression enhanced the binding of EZH2 to the promoter of the DIRAS3. **M** and **N** The ChIP assay demonstrated that diminishing of RNF157-AS1 decreased the binding of HMGA1 to the promoter of ULK1 genes and RNF157-AS1 overexpression enhanced the binding of HMGA1 to the promoter of ULK1. Data were obtained from at least three independent experiments. *P < 0.05; **P < 0.01; ***P < 0.001 (*t*-test).

To further assess the downstream genes of RNF157-AS1, RNA transcriptome sequencing (RNA-seq) was used in SKOV3 cells with siRNA-mediated suppression of RNF157-AS1. A common set of 24 mRNAs exhibited altered expression (Fig. 4A, B) (fold changes ≥ 2, FDR < 0.05). Among them, DIRAS3 has been reported to have an important role in EOC cells and is regulated by EZH2 [25]. From the GEPIA database, DIRAS3 expression showed a negative correlation with RNF157-AS1 expression (Fig. 4C). In addition, previous studies have reported that HMGA1 binds to the promoters of FOXM1 [26], FOXO1 [27], ULK1 [28], MMP2 [29], KL [30], and then promote or suppress target gene expression. The RNA-seq result indicated that ULK1 increased ~1.23-fold after RNF157-AS1 knockdown (data not shown). In the GEPIA database ULK1 expression had a negative correlation with RNF157-AS1 expression (Fig. 4D). Therefore, whether DIRAS3 and ULK1 are the targets of RNF157-AS1 and EZH2 was our primary focus. We next investigated the mRNA and protein expression levels of DIRAS3 and ULK1 after overexpression or knockdown of RNF157-AS1. The results suggested that RNF157-AS1 knockdown enhanced the expression of DIRAS3 and ULK1 while RNF157-AS1 overexpression inhibited DIRAS3 and ULK1 expression (Fig. 4E–J). Next, the ChIP-qPCR assay results revealed that inhibiting RNF157-AS1 reduced the binding of EZH2 to the promoter of DIRAS3 and the binding of HMGA1 to the promoter of ULK1 while overexpression of RNF157-AS1 increased the binding of EZH2 to the promoter of DIRAS3 and the binding of HMGA1 to the promoter of ULK1 (Figs. 4K–N and S3).

### RNF157-AS1 inhibited the autophagy of EOC cells through DIRAS3 and ULK1

**Fig. 5:**
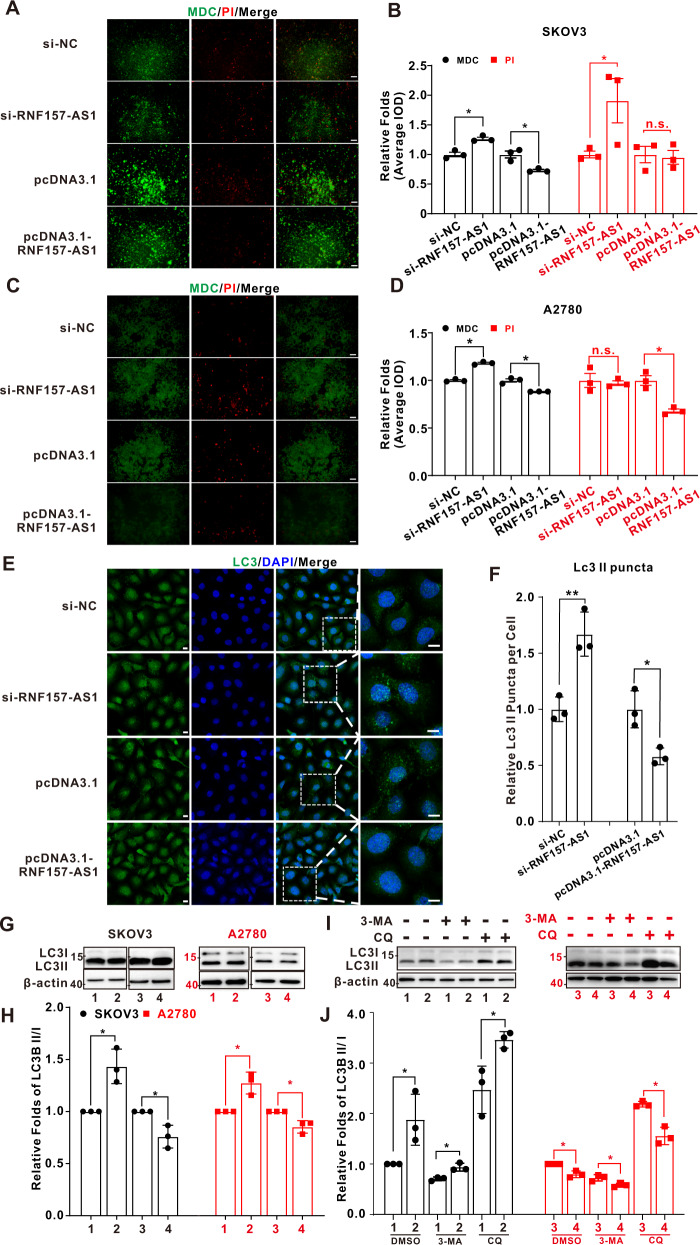
RNF157-AS1 inhibited autophagy in EOC cells. **A** MDC/PI dual staining in SKOV3 cells. The bar represents 100 μm. **B** Quantitative analysis of the IOD indicated RNF157-AS1 knockdown increased the MDC staining and RNF157-AS1 overexpression decreased MDC staining, but there was no significant difference in PI staining. **C** Autophagy/cytotoxicity dual staining in A2780 cells. The bar represents 100 μM. **D** Quantitative analysis of the MDC or PI staining indicated RNF157-AS1 knockdown increased autophagy level and RNF157-AS1 overexpression decreased autophagy level, but it had no effect on apoptosis. **E** LC3 staining in SKOV3 cells after RNF157-AS1 knockdown or overexpression. The bar represents 10 μm. **F** Quantitative analysis of the relative number of LC3 puncta (LC3 II) in every cell. **G** The LC3 II and LC3 I expression was detected by western blot in SKOV3 cells and A2780 cells after RNF157-AS1 knockdown or overexpression. Full-length blots are presented in Supplementary Fig. S7. **H** Quantitative analysis of the ratio of LC3 II/LC3 I in SKOV3 cells and A2780 cells after RNF157-AS1 knockdown or overexpression (1: si-NC; 2: si-RNF157-AS1; 3: pcDNA3.1; 4: pcDNA3.1-RNF157-AS1). **I** Western blot analysis of the protein from A2780 cells transfected with RNF157-AS1 interference or overexpression plasmid and then treated with 3-MA (100 μM, M9281, Sigma) or CQ (100 μM, C6625, Sigma) for 6 h. Full-length blots are presented in Supplementary Fig. S8. **J** Quantitative analysis of the ratio of LC3 II/LC3 I/β-actin (1: si-NC; 2: si-RNF157-AS1; 3: pcDNA3.1; 4: pcDNA3.1-RNF157-AS1). Data were obtained from at least three independent experiments. *P < 0.05; **P < 0.01 (*t*-test).

Both DIRAS3 and ULK1 are pivotal autophagy initiation proteins. Whether RNF157-AS1 affects EOC cell autophagy is still unclear. The results of autophagy/cytotoxicity dual staining indicated that RNF157-AS1 overexpression decreased monodansylcadaverine (MDC) staining and RNF157-AS1 inhibition enhanced the MDC staining in SKOV3 and A2780 cells. However, RNF157-AS1 had no effect on apoptosis (Fig. 5A–D). Since LC3 is a key marker of autophagy, we further examined the LC3 I and LC3 II level. The level of LC3 II (relative LC3 puncta per cell) declined significantly when RNF157-AS1 was overexpressed and increased when RNF157-AS1 was knocked down according to immunofluorescence (IF) assay results (Fig. 5E, F), and this pattern was confirmed by western blot, suggesting that RNF157-AS1 decreased the LC3 II/I ratio (Fig. 5G, H). Therefore, MDC staining and the LC3 II/I ratio were consistent in indicating that RNF157-AS1 suppressed autophagy in EOC cells. To further prove whether RNF157-AS1 affects the direction of autophagic flux, the autophagy inhibitor 3-methyladenine (3-MA), and chloroquine (CQ) were used according to previous study [31]. As shown in Fig. 5I and J, 3-MA treatment decreased the LC3 II levels while CQ treatment increased the 3II levels. Moreover, the ratio of LC3 II/I between si-NC and si-RNF157-AS1 or between pcDNA3.1 and pcDNA3.1-RNF157-AS1 became larger in the CQ treatment group than DMSO and 3-MA treatment group. These results suggested that LC3 II accumulation is indeed caused by the altering of RNF157-AS1.

### RNF157-AS1 reduced the resistance of EOC cells to extracellular stress through the suppression of autophagy

**Fig. 6:**
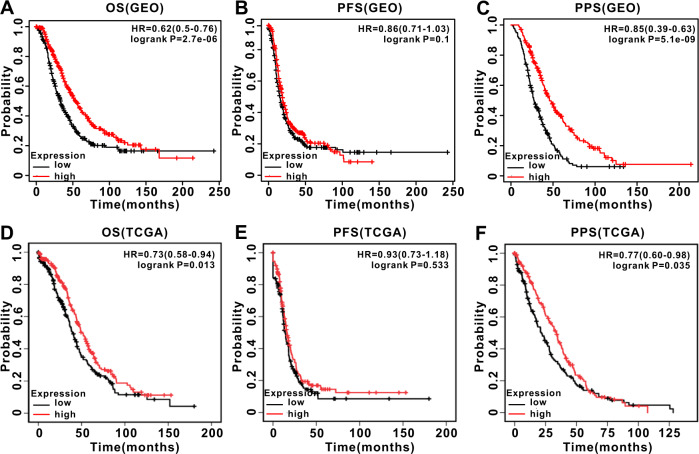
RNF157-AS1 overexpression indicated a favorable prognosis for EOC patients based on GEO and TCGA databases. According *Kaplan–Meier Plotter*, EOC patients in the GEO database (including GSE18520, GSE19829, GSE26193, GSE27651, GSE30161, GSE63885, and GSE9891) with high expression of RNF157-AS1 had a good prognosis for OS (**A**) and PPS (**C**) prognoses, but not a good for PFS prognosis (**B**) compared with those patients with low expression of RNF157-AS1. Based on TCGA database analysis, EOC patients with high RNF157-AS1 exhibited increased OS (**D**) and PPS (**F**) time compared with patients with low levels of RNF157-AS1 expression. However, RNF157-AS1 expression had no effect on the PFS (**E**) time.

We next examined the association of RNF157-AS1 expression with clinicopathological features of EOC patients. A total of 416 EOC patients with complete primary clinical data in the TCGA database were used for the analysis. As shown in Table 1, RNF157-AS1 expression was not associated with any primary clinicopathological features. We further used Cox regression analysis to determine the independent prognostic markers in EOC patients. By univariate analysis, RNF157-AS1 was identified as an independent prognostic factor for overall survival (OS) and post-progression survival (PPS) but not for progression-free survival (PFS) (Table 2). Multivariate analysis also suggested that RNF157-AS1 was an independent prognostic factor for OS. Moreover, according to the *Kaplan‒Meier Plotter* (http://kmplot.com/analysis) database (EOC patients from GSE18520, GSE19829, GSE26193, GSE27651, GSE30161, GSE63885, and GSE9891) and TCGA database, high expression of RNF157-AS1 were associated with shorter OS times and shorter PPS times than was the low expression of RNF157-AS1 in EOC patients in both the GSE database and TCGA database. However, RNF157-AS1 expression had no effect on the PFS of EOC patients (Fig. 6).

**Table 1:** Clinicopathological features of RNF157-AS1 in EOC patients based on TCGA.

Clinicpathological features	No. of patients	RNF157-AS1 expression		*P*^a^
		Low group (*N* = 208)	High group (*N* = 208)	
*Age*	416			0.623
<60		108(48.9%)	113(51.1%)	
≥60		100(51.3%)	95(48.7%)	
*FIGO stage*	413			0.387
I/II		9(40.9%)	13(59.1%)	
III/IV		197(50.4%)	194(49.6%)	
*Grade*	408			0.381
G1/G2		30(55.6%)	24(44.4%)	
G3/G4		174(49.2%)	180(50.8%)	
*Site*	391			0.312
Left/Right		55(55.0%)	45(45.0%)	
Bilateral		143(49.1%)	148(50.9%)	
*Disease free status*	351			0.881
Disease free		42(48.3%)	45(51.7%)	
Recurred/progressed		125(47.3%)	139(52.7%)	
*Lymphatic invasion*	159			0.786
No		25(46.3%)	29(53.7%)	
Yes		51(48.6%)	54(51.4%)	
*Neoplasm status*	366			0.47
Tumor free		42(45.7%)	50(54.3%)	
With tumor		137(50.0%)	137(50.0%)	
*Overall survival status*	416			0.363
Living		134(51.7%)	125(48.3%)	
Deceased		74(47.1%)	83(52.9%)	

^a^*p*-values were determined by using the Chi-squared test.

**Table 2:** Univariate and multivariate Cox regression analysis RNF157-AS1 for OS, PFS, or PPS of EOC patients in TCGA database.

Variables	OS			PFS			PPS		
	HR	95% CI	*P*	HR	95% CI	*P*	HR	95% CI	*P*
*Univariate analysis*									
Age (<60 vs. ≥60)	**1.371**	**1.074–1.749**	0.011*	1.132	0.888–1.444	0.317	**1.321**	**1.033–1.689**	0.027*
TNM stage (I/II vs. III/IV)	1.958	0.923–4.157	0.080	1.279	0.980–1.668	0.070	1.433	0.675–3.041	0.349
Grade (G1/G2 vs. G3/G4)	1.139	0.802–1.616	0.467	1.055	0.765–1.455	0.745	1.203	0.844–1.715	0.306
Site (unilateral vs. bilateral)	1.070	0.799–1.434	0.648	1.109	0.858–1.435	0.428	1.029	0.768–1.378	0.849
Neoplasm status (tumor free vs. with tumor)	**7.912**	**4.501–13.907**	<0.001*	**5.578**	**3.816–8.152**	<0.001*	**3.882**	**2.212–6.815**	<0.001*
Lymphatic invasion (no vs. yes)	1.162	0.717–1.882	0.543	1.122	0.750–1.679	0.575	0.909	0.558–1.482	0.703
RNF157-AS1 expression (low vs. high)	**0.734**	**0.575–0.937**	0.013*	0.926	0.727–1.180	0.533	**0.769**	**0.601–0.983**	0.036*
*Multivariate analysis*									
Age(<60 vs. ≥60)	1.267	0.976–1.645	0.075	–	–	–	1.253	0.963–1.630	0.093
Neoplasm status (tumor-free vs. with tumor)	**7.919**	**4.504–13.924**	<0.001*	**5.578**	**3.816–8.152**	<0.001*	**3.97**	**2.260–6.975**	<0.001*
RNF157-AS1 expression (low vs. high)	**0.72**	**0.554–0.935**	0.014*	–	–	–	0.771	0.593–1.003	0.053

The bold values indicated these data are statistically different, including HR, 95% CI and *P* value.

*P < 0.05.

**Fig. 7:**
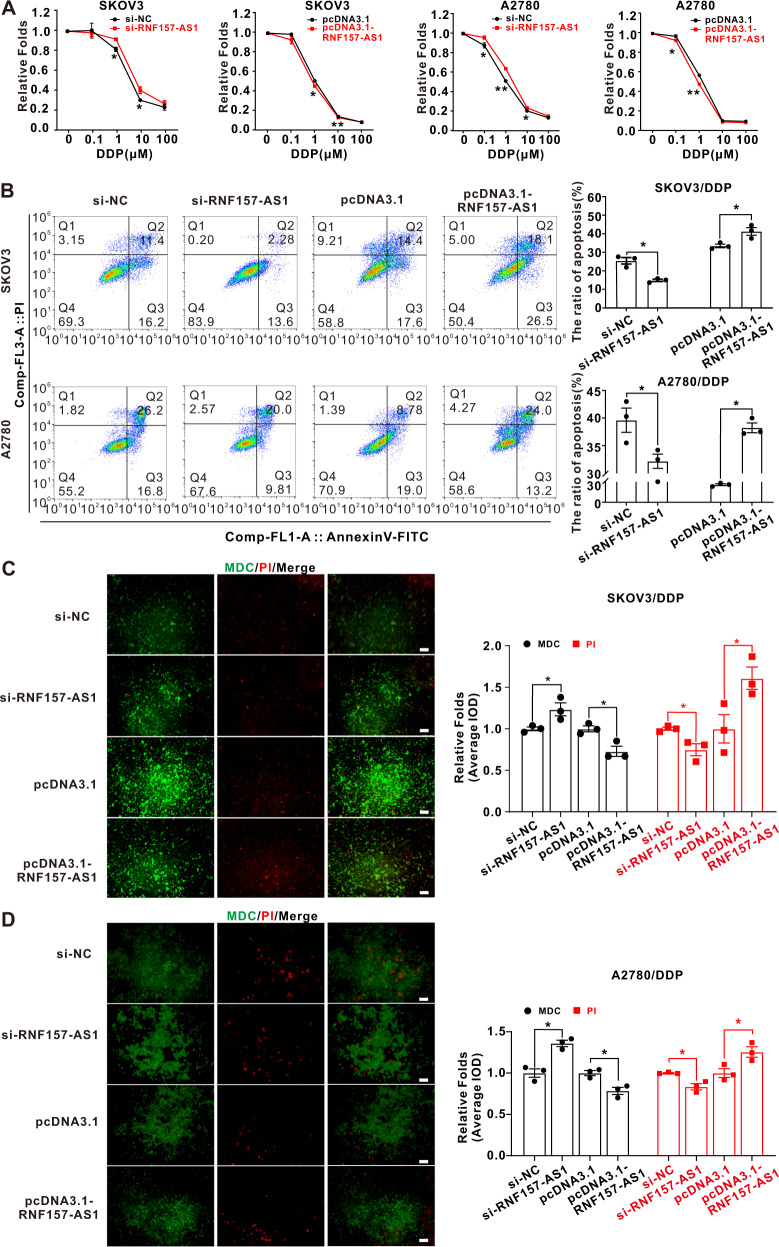
RNF157-AS1 reduced the viability and promoted the apoptosis of EOC cells under a DDP environment. **A** Treatment with DDP (2 μM) for 48 h, RNF157-AS1 overexpression significantly reduced SKOV3 and A2780 cell viability but RNF157-AS1 depletion enhanced EOC cell viability. **B** Treatment with DDP (2 μM) for 48 h, RNF157-AS1 interference markedly reduced the apoptosis rate of SKOV3 and A2780 cells and RNF157-AS1 overexpression increased the apoptosis rate of EOC cells. **C** and **D** Under the DDP environment, RNF157-AS1 depletion increased MDC staining and decreased IP staining. on the contrary, RNF157-AS1 overexpression elevated MDC staining and diminished IP staining. The bar represents 100 μm. Data were obtained from at least three independent experiments. *P < 0.05; **P < 0.01 (*t*-test).

Why is RNF157-AS1 overexpressed in EOC tissues compared with normal ovarian tissues, but EOC patients with RNF157-AS1 overexpression have a better prognosis? We speculated that RNF157-AS1 attenuates autophagy and results in intolerance to extracellular stress in EOC cells. Because platinum-based chemotherapy is still the first-line treatment for EOC patients, we determined the effect of RNF157-AS1 on cis-platinum (DDP)-resistance in EOC cells. The results showed that RNF157-AS1 overexpression promoted DDP-resistance in EOC cells and inhibited SKOV3 and A2780 cell apoptosis after treatment with DDP. In contrast, the knockdown of RNF157-AS1 enhanced apoptosis and attenuated resistance to DDP in EOC cells (Fig. 7A, B). We further exploited MDC/PI staining to elucidate the relationship between autophagy and DDP-resistance in EOC cells. As shown in Fig. 7C and D, under the DDP environment, RNF157-AS1 overexpression decreased autophagy and promoted apoptosis but RNF157-AS1 inhibition enhanced autophagy and suppressed apoptosis in SKOV3 and A2780 cells.

### Different effects of RNF157-AS1 on EOC under different environments by autophagy in vivo

**Fig. 8:**
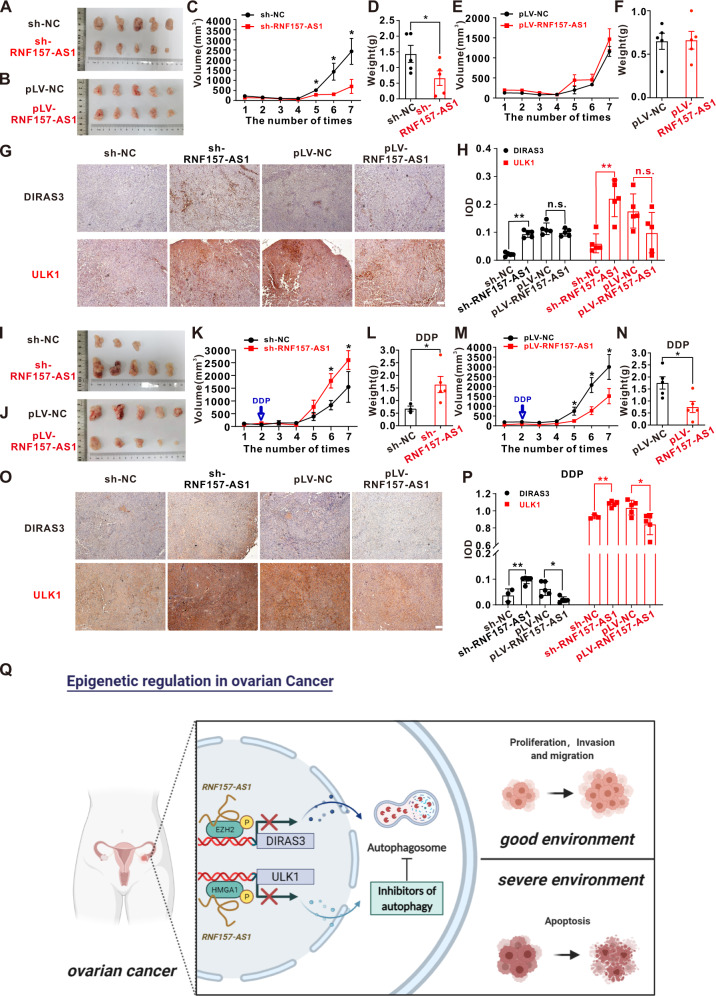
RNF157-AS1 exerts differential effects on EOC in different environments by suppressing autophagy in vivo. **A** sh-NC and sh-RNF157-AS1 were stably transfected into A2780 cells and injected in the nude mice. **B** pLV-NC and pLV-RNF157-AS1 were stably transfected into A2780 cells and injected in the nude mice. **C** and **D** The tumor volumes and the tumor weights of sh-NC and sh-RNF157-AS1 were calculated. The bars indicated SEM. **E** and **F** The tumor volumes and the tumor weights of pLV-NC and pLV-RNF157-AS1 were calculated. The bars indicated SEM. **G** and **H** IHC staining of DIRAS3 and ULK1 in the tumor with stably transfecting sh-NC, sh-RNF157-AS1, pLV-NC, and pLV-RNF157-AS1. **I** After being injected with A2780 cells with stably transfecting sh-NC and sh-RNF157-AS1 for 2 weeks, the mice were treated with DDP twice a week. **J** pLV-NC and pLV-RNF157-AS1 were stably transfected into A2780 cells and injected in the nude mice for 2 weeks, the mice were treated with DDP twice a week. **K** and **L** The tumor volumes and the tumor weights of sh-NC and sh-RNF157-AS1 were calculated after treatment with DDP. The bars indicated SEM. **M** and **N** The tumor volumes and the tumor weights of pLV-NC and pLV-RNF157-AS1 were calculated after treatment with DDP. The bars indicated SEM. **O** and **P** IHC staining of DIRAS3 and ULK1 in the tumor with stably transfecting sh-NC, sh-RNF157-AS1, pLV-NC, and pLV-RNF157-AS1 after treated with DDP. **Q** Model explaining the mechanism of RNF157-AS1 in EOC cells in different environments. Data were obtained from at least three independent experiments. *P < 0.05; **P < 0.01 (*t*-test).

To identify the effect of RNF157-AS1 on EOC cells, we injected SKOV3 cells into nude mice after transfecting them with or without siRNA against RNF157-AS1. As shown in Fig. S4, compared with that in the negative control group, the knockdown of RNF157-AS1 significantly suppressed tumor growth. A2780 cells were also stably transfected with sh-RNF157-AS1, pLV-RNF157-AS1, or the corresponding control vector. Then, these stably transfected cells were inoculated into nude mice. Twenty-four days after injection, the tumors formed in the sh-RNF157-AS1 group were substantially smaller than those in the control group. However, although there were no significant differences, the tumor size and weight of the pLV-RNF157-AS1 group were slightly greater than those in the control group (Fig. 8A–F). Consistent with the tumor size and weight, DIRAS3 and ULK1 expression in tumor tissues were significantly overexpressed in tumor tissues in the sh-RNF157-AS1 group compared with the sh-NC group, while there was no significant difference between the pLV-RNF157-AS1 and pLV-NC groups in the expression of DIRAS3 and ULK1 in tumor tissues (Fig. 8G, H). We then sought to further validate the effect of RNF157-AS1 on EOC tumors under the DDP environment. As shown in Fig. 8I–N, the tumor size and weight in the sh-RNF157-AS1 group were substantially greater than those in the control group while the tumors formed in the pLV-RNF157-AS1 group were significantly smaller than those in the pLV-NC group after treatment with DDP. However, DIRAS3 and ULK1 expression was upregulated in the sh-RNF157-AS1 group and downregulated in the pLV-RNF157-AS1 group compared to the control group (Fig. 8O, P). On balance, this in vivo assay still sheds light on the role of RNF157-AS1-mediated autophagy in different environments.

In conclusion, RNF157-AS1 serves as a scaffold binding the EZH2 and HMGA1 proteins and regulates DIRAS3 and ULK1 expression in EOC cells. ULK1 and DIRAS3 are two important autophagy initiation proteins. RNF157-AS1-mediated autophagy promotes EOC cell proliferation, invasion, and migration in a good environment for survival. However, RNF157-AS1-mediated inhibition of autophagy enhances EOC cell apoptosis in harsh environments (Fig. 8Q).

## Discussion

The lncRNA RNF157-AS1 was found to be overexpressed in EOC tissues compared with normal ovarian tissues in many studies. The role and mechanism of RNF157-AS1 in cancer, however, are comparatively poorly understood. In our study, RNF157-AS1 did not affect apoptosis, but it did boost EOC cell proliferation, invasion, and migration in a good environment. Mechanistic experiments revealed that RNF157-AS1 can bind to EZH2 and HMGA1, acting as a modular scaffold to inhibit the autophagy genes DIRAS3 and ULK1. RNF157-AS1 consequently attenuated autophagy in EOC cells. However, RNF157-AS1-mediated autophagy inhibition enhanced the DDP sensitivity of EOC cells. Hence, RNF157-AS1 could be used as an indicator of a favorable prognosis for EOC patients. To the best of our knowledge, our study is the first to describe the function and mechanism of RNF157-AS1.

Is the lncRNA RNF157-AS1 beneficial or harmful for EOC patients? Lu Z. et al. suggested that DIRAS3 could regulate autophagy and ovarian cancer cells’ dormancy [4, 19]. In dormant ovarian cancer cells, DIRAS3 likewise controls the autophagosome initiation complex [5]. Therefore, DIRAS3-mediated autophagy could promote ovarian cancer cell dormancy. In our study, although inhibition of RNF157-AS1 suppressed the proliferation, invasion, and migration of EOC cells, RNF157-AS1 had no effect on apoptosis. The reason is that RNF157-AS1 knockdown might promote EOC cell dormancy by regulating autophagy. The proliferation, invasion, and migration of dormant EOC cells were suppressed but apoptosis was unaffected. However, in a harsh environment, such as during exposure to chemotherapy, dormant cells are resistant to extracellular stress. Platinum-based chemotherapy is the recommended course of treatment for those with advanced EOC because the majority of individuals with this condition lose the option of surgical resection. Therefore, EOC patients with high RNF157-AS1 expression have longer OS and PPS times than patients with low RNF157-AS1 expression. Therefore, RNF157-AS1 is a good prognostic indicator for EOC patients.

To further explore the molecular mechanism underlying RNF157-AS1 in EOC cells, we investigated the regulation of DIRAS3 and ULK1 by RNF157-AS1. This is the first study revealing the inhibitory effect of RNF157-AS1 on DIRAS3 and ULK1 expression. In this study, DIRAS3 overexpression-mediated by RNF157-AS1 suppression decreased the proliferation, invasion, and migration of EOC cells but had no significant effect on apoptosis. This finding is consistent with a previous study suggesting that DIRAS3-induced autophagy resulted in reversible cell death under conditions of transient DIRAS3 overexpression in vitro [6]. However, in our study, RNF157-AS1-mediated DIRAS3- and ULK1-related autophagy enhanced the survival of EOC cells under the DDP environment. This result was in contrast to that of Lu et al., suggesting that DIRAS3-mediated autophagy-associated cell death enhances the chemosensitivity of EOC cells [32]. That is because the different effects are likely due to RNF157-AS1-mediated autophagy not only through DIRAS3 but also through ULK1. In addition, Lu and colleagues’ results showed that DIRAS3-induced autophagy contributed to cell survival and tumor dormancy in an ovarian cancer xenograft model, which also supported our findings. Of course, maximal induction of autophagy by DIRAS3 overexpression might result in irreversible autophagic cell death. However, lncRNAs display lower expression levels than protein-coding genes. Hence, in our study, it was impossible for DIRAS3 to be maximally induced by RNF157-AS1 knockdown. Therefore, our data show that RNF157-AS1-mediated autophagy via suppression of DIRAS3 and ULK1 expression might lead to different outcomes in different environments.

In our study, RNF157-AS1 was identified as a favorable prognostic indicator for OS and PPS but not for PFS in individuals with EOC. PPS is often used as the primary efficacy endpoint in most clinical trials of cancer, especially after on-trial cancer progression [33, 34]. PFS indicates the endpoint of temporary remission of tumors and does not represent the final outcome of cancer patients. Thus, OS and PPS are more clinically relevant clinically. In our study, RNF157-AS1 was associated with autophagy by inhibiting DIRAS3 and ULK1 expression in EOC cells. Autophagy is the cellular process that occurs when parts of cell components are engulfed in response to a severe environment. In a good environment, autophagy was decreased in EOC cells. Moreover, as shown in Fig. 2 in our study, the occurrence of autophagy may even reduce the cell proliferation rate. Therefore, RNF157-AS1 was not related to PFS in EOC patients because autophagy did not play any role in the nonprogressive stage of the tumor. On the contrary, in a severe environment, more autophagosomes are formed in the cells to increase cell survival. Autophagy plays a striking role in EOC cell survival after advanced tumor progression or DDP chemotherapy. Therefore, in our study, RNF157-AS1 overexpression predicted longer PPS and even OS times of EOC patients through the inhibition of autophagy.

In our mRNA-seq results, there was no striking difference in ULK1 expression between the si-NC and si-RNF157-AS1 groups. We chose ULK1 as the target gene of HMGA1 for the following reasons. In previous studies, FOXM1, FOXO1, ULK1, MMP2, and KL were reported to be regulated by HMGA1. These five genes were selected as the candidate target genes of HMGA1 and RNF157-AS1. In our raw mRNA-seq data, only FOXM1 (1.26-fold change, *P* = 0.016 < 0.05) and ULK1 (1.23-fold change, *P* = 0.001 < 0.05) were upregulated in si-RNF157-AS1 compared with si-NC. However, Zanin R. et al. reported HMGA1 positively promoted the stability, nuclear localization, and transcriptional activity of FOXM1 and strengthened tumor angiogenesis [26]. In addition, Andrea Conte and colleagues suggested that HMGA1 negatively regulates ULK1 transcription [28]. Thus, collectively, previous studies reports and our mRNA-seq indicated that HMGA1 coupled with RNF157-AS1 were more likely to regulate ULK1 in our study. What’s more, RNF157-AS1 knockdown significantly depressed the mRNA and protein expression levels of ULK1 in both EOC cells and tumors. Finally, RNF157-AS1 interacts with HMGA1 bound to the promoter of ULK1 and negatively regulated ULK1 expression and autophagy in EOC cells.

In our RNA pulldown results, 17 proteins, including HMGA1, were detected in the sense group (RNF157-AS1 group). However, EZH2 was not detected in either the sense group or the antisense group (control group). We thought that this was related to the sensitivity of LC–MS/MS. Although the sensitivity of LC–MS/MS is very high, it depends on the expression levels of other proteins. High concentrations of proteins obscure low concentrations of proteins and lead to the inability to detect low concentrations of protein by LC–MS/MS. This is why the protein with the highest levels must be removed when detecting serum proteins by LC–MS/MS. In addition to LC–MS/MS, we also used western blot to verify the RNA pulldown result. In our study, we found not only HMGA1 but also EZH2 in the RNF157-AS1 group. That is because the western blot only detected one protein by the cascade amplification method without interference from high-concentration proteins. This is the reason that HMGA1 also was detected not only in the sense group but also in the antisense group. However, the different level of HMGA1 between the two groups was very large, which still indicated that RNF157-AS1 could bind to the HMGA1 protein.

In our study, we utilized two methods and two cell lines to explore the effect of RNF157-AS1 on the growth of EOC in vivo. First, we injected SKOV3 cells into nude mice and then injected intratumorally siRNA once a week. Although siRNA cannot stably knockdown RNF157-AS1 in EOC tumors, this intertumoral injection method is still used in tumor-bearing nude mice [35, 36]. Then we also used shRNA or overexpression virus vector to generate stable A2780 cells. Injection of these stable cells still indicated that RNF157-AS1 promotes EOC tumor growth. We used A2780 cells not SKOV3 cells as stable cells because A2780 cells are prone to form tumors. In addition, the stable A2780 cells had a reduced risk of nontumorigenesis after DDP treatment. In brief, different cells and different methods have important effects on the growth and tumor progression of EOC.

The lncRNA RNF157-AS1 might often be ignored by researchers because it mediates autophagy and has different roles under different environments. Mechanistically, RNF157-AS1 not only binds to EZH2 to suppress DIRAS3 expression but also binds to HMGA1 to inhibit ULK1 expression. Functionally, RNF157-AS1 can enhance EOC cell viability by repressing autophagy in a good environment while it also reduces the growth of EOC cells to adapt to a harsh environment by negatively regulating autophagy. Therefore, the lncRNA RNF157-AS1 participates in the occurrence and development of EOC, although it has the opposite roles in different stages. Our study not only provides a new treatment strategy for advanced EOC but also provides a perspective for the re-examination lncRNAs that were found to have functions in different studies.

## Materials and methods

### Cells culture

EOC cells SKOV3, A2780, and OVCAR3 were purchased from the Cell bank of the Chinese Academy of Sciences (Shanghai, China). SKOV3 cells were cultured in McCoy’s 5A (KeyGen, Nanjing, China) with 10% FBS (Gibco, Grand Island, NY), A2780 cells were cultured in DMEM with high glucose (Gibco, Grand Island, NY) with 10% FBS and OVCAR3 cells were cultured in 1640 medium (Gibco) with 20% FBS.

### Cell transfection of interference and vectors

SKOV3 and A2780 cells were transfected with RNF157-AS1-specific siRNAs (Invitrogen) and RNF157-AS1 overexpression vector based on pcDNA3.1 (Genscript Biotech, Nanjing, China) with Lipofectamine 3000 (Invitrogen) and 10 nM siRNA in Opti-MEM (Invitrogen). The siRNA of RNF157-AS1 sequences is shown in Table S1.

### RNA extraction and qPCR

Total RNA extraction and reverse transcription were conducted by Thermo Scientific GeneJET RNA Purification Kit (Thermo Scientific, MA, USA) and RevertAid First-Strand cDNA Synthesis Kit (Thermo Scientific). Then qPCR was performed using SYBR Green. The primer for RNF157-AS1 was reported in our previous study [22]. The primers of DIRAS3 and ULK1 were synthesized according to Fu Y. et al. [25] and Conte A. et al. [28]. The primers of 45S and 12S, nuclear or cytosolic markers, were synthesized according to a previous study [37]. All the primer sequences are in Table S3.

### Subcellular fractionation location

RNA nuclear and cytosolic were separated using a PARIS Kit (Life Technologies, CA, USA) according to previous studies [38].

### Cell proliferation assays

The viability of EOC cell proliferation was tested using a CCK8 kit (KeyGen Biotech Co. Ltd.) and clone formation assay. The CCK8 was performed after transfection with the RNF157-AS1 siRNA or overexpression vector. Cells were incubated at 37 °C for 1 h with CCK8 reagent after culture for 0, 24, 48, and 72 h. The reference wavelength of 450 nm was used to determine the OD value. For the clone formation assay, ~1000–3000 SKOV3 and A2780 cells after transfected with siRNA or vector were seeded in six-well plates. After ~2 weeks, the cells were fixed and stained with 4% paraformaldehyde and stained with crystal violet. The colony number was determined and analyzed by Image J software (NIH, MD, USA).

### Flow-cytometric analysis

Transfected EOC cells were harvested after 48 h and then the cell supernatant and washed cold PBS was collected in a tube. Cells were digested using EDTA-free trypsin (Invitrogen) and were collected in a tube. The collected cells were treated with an Annexin V/PI kit (Vazyme Biotech Co., Ltd, Nanjing, China) according to the manufacturer’s instructions and analyzed by flow cytometry (Beckman, FL, USA).

### Transwell invasion and migration assays

Cell invasion and migration assays were performed using the 24-well Transwell chambers (8 μm, Corning, NY, USA). For the cell invasion assay, the 24-well cell culture insert surface was coated with Matrigel (BD, CA, USA). Transfected EOC cells in medium containing 5% FBS were placed in the top chamber and medium with 10% FBS was filled into the bottom chambers. After 72 h, the invaded and migrated cells were fixed and stained. The number of cells was analyzed by Image J software (NIH).

### Autophagy/cytotoxicity dual staining assay

MDC and PI staining were performed using an autophagy/cytotoxicity dual staining Kit (ab133075, Abcam, MA, USA) according to the manufacturer’s instructions. Images were acquired with a microscope (100×, Zeiss, Jena, Germany). Then, the integrated optical density (IOD) of MDC and PI were analyzed by Image Pro Plus software 6.0 (Silver Springs, MD, USA).

### Western blot

Proteins from transfected cells were extracted with RIPA lysis buffer (Beyotime, China) supplemented with PMSF (Beyotime, China). The proteins were separated by 10–15% SDS–polyacrylamide gel electrophoresis (SDS–PAGE). Then the proteins were transferred to PVDF membranes and incubated with specific antibodies. Anti-EZH2(5246S), anti-LC3B (3868S), and anti-β-actin (4970S) antibodies were purchased from Cell Signaling Technology (Boston, MA, USA). Anti-HMGA1 (ab252930) and anti-DIRAS3 (ab107051) antibodies were purchased from Abcam (Cambridge, MA, USA). Anti-ULK1 (20986-1-AP) and anti-α-tubulin (11224-1-AP) antibodies were purchased from ProteinTech. Band densities were quantified by Image J software (NIH, MD, USA).

### Immunofluorescence assay

SKOV3 cells were grown on thick slides in a 24-well plate. After transfection for 48 h, cells were washed and fixed with 4% polyformaldehyde for 20 min. Then the cells were permeabilized with 0.1% Triton-X-100 and blocked with bovine serum albumin (BSA). SKOV3 cells were incubated with an anti-LC3B monoclonal antibody (3868S) overnight at 4 °C and then followed by incubation with an anti-rabbit Alexa Fluor 488-conjugated secondary antibody (The Jackson Laboratory, Bar Harbor, ME, USA). Subsequently, SKOV3 cells were stained with DAPI for 2 min. Coverslips and cells were captured and analyzed by Confocal Laser Scanning Microscopy (×630, Leica, Wetzlar, Germany).

### RNA pulldown

The in vitro RNA pulldown assay with a biotin-coupled probe was performed by GeneCreate Biotech (Wuhan, China) with Pierce^TM^ Magnetic RNA-protein Pull-Down Kit (#20164, Thermo Fisher Scientific, Waltham, MA, USA) according to the manufacturer’s instructions. Then, the eluted proteins from the sense and antisense groups were used for mass spectrometry analysis.

### RNA immunoprecipitation assay

The RNA immunoprecipitation (RIP) assay was performed using a Magna RIP^TM^ RNA-Binding Protein Immunoprecipitation Kit (Millipore, Billerica, MA, USA) according to the manufacturer’s instructions. ChIP grade antibodies against EZH2 (ab191250) and HMGA1(ab252930) were from Abcam.

### Chromatin immunoprecipitation (ChIP)-qPCR assays

The ChIP assays were performed using EZ-ChIP Kit (Millipore, Billerica, MA, USA) according to the manufacturer’s instructions. ChIP grade antibodies for EZH2 (ab191250) and HMGA1(ab252930) were from Abcam. The ChIP primer of the promoter region of DIRAS3 [25] and ULK1 [28] can be found in Supplementary Table 3 (Table S3) based on previous studies reported. Quantification of immunoprecipitated DNA was performed using qPCR.

### RNA-seq

SKOV3 cells were transfected with si-NC and si-RNF157-AS1 for 48 h and washed twice. Then 1 ml of TRIzol (Invitrogen) was added. The RNA-seq was performed by GENEWIZ (Suzhou, China).

### Xenograft study

For transient transfection cells, the fourteen 6-week-old BALB/c nude mice were randomly divided into two groups (seven mice per group). SKOV3 cells were transiently transfected with si-NC or si-RNF157-AS1 using Lipofectamine 3000, After 24 h, ~2 × 10^6^ cells were injected subcutaneously into nude mice. To maintain the interference efficiency, about 20 μg of siRNA per mouse was injected once a week. Tumor volume and mice weight was measured once a week. For stable transfection cells, the twenty 6-week-old BALB/c nude mice were randomly divided into four groups (five mice per group). A2780 cells were transfected by pLV-NC/pLV-RNF157-AS1 (Cyagen, Suzhou, China) or sh-NC/sh-RNF157-AS1(GenePharma, Shanghai, China). Then the stable transfection cells 2 × 10^6^ cells were injected subcutaneously into 6-week-old female nude mice. Tumor volume and mice weight were measured twice a week. For DDP treatment, every mouse in the DDP-treated group was treated with DDP (5 mg/kg) twice a week by intraperitoneal injection beginning 2 weeks after cell injection based on a previous study [39]. Tumor volume (*V*) was monitored by measuring the length (*L*) and width (*W*) with calipers and was calculated with the formula 0.5 × *L* × *W*^2^. All animal experiments were conducted in accordance with the institutional standard guidelines of Nanjing Medical University.

### IHC

DIRAS3 and ULK1 protein expression in tumor tissues was assessed by IHC Kit (PK10086, ProteinTech) according to the manufacturer’s protocol. Deparaffinized sections were boiled in citrate buffer for antigen retrieval and then incubated with anti-DIRAS3 (ab107051, Abcam) and anti-UIK1(20986-1-AP, ProteinTech) antibodies. Selected regions were captured under a light microscope and the IOD of DIRAS3 and ULK1 protein expression were analyzed by IPP 6.0 software.

### Statistical analysis

Repeated experiments were performed to minimize statistical variance. All data were subjected to statistical analysis. Two-tailed Student’s *t*-test and the chi-square test were performed using SPSS 20.0 statistical software (SPSS, USA). Data were obtained from at least three independent experiments and were presented as means ± standard deviation (SD), the data of animals were presented as means ± standard error of the mean (SEM), and clinical data are shown as the rate (%). Differences were considered significant when the *P* value was <0.05.

## Supplementary information

Figure S1

Figure S2

Figure S3

Figure S4

Figure S5

Figure S6

Figure S7

Figure S8

Table S1

Table S2

Table S3

Supplemental figure legend

AJ-checklist

## Data Availability

The raw data of this study are available on Sequence ReadArchive (SRA) database: https://www.ncbi.nlm.nih.gov/sra/PRJNA856726.
