# Supplementary figures and images for: Differential effects of the LncRNA RNF157-AS1 on epithelial ovarian cancer cells through suppression of DIRAS3- and ULK1-mediated autophagy
Source: Cell Death Dis. 2023 Feb 20;14(2):140. doi: 10.1038/s41419-023-05668-5 (PMC9941098; doi:10.1038/s41419-023-05668-5)

A

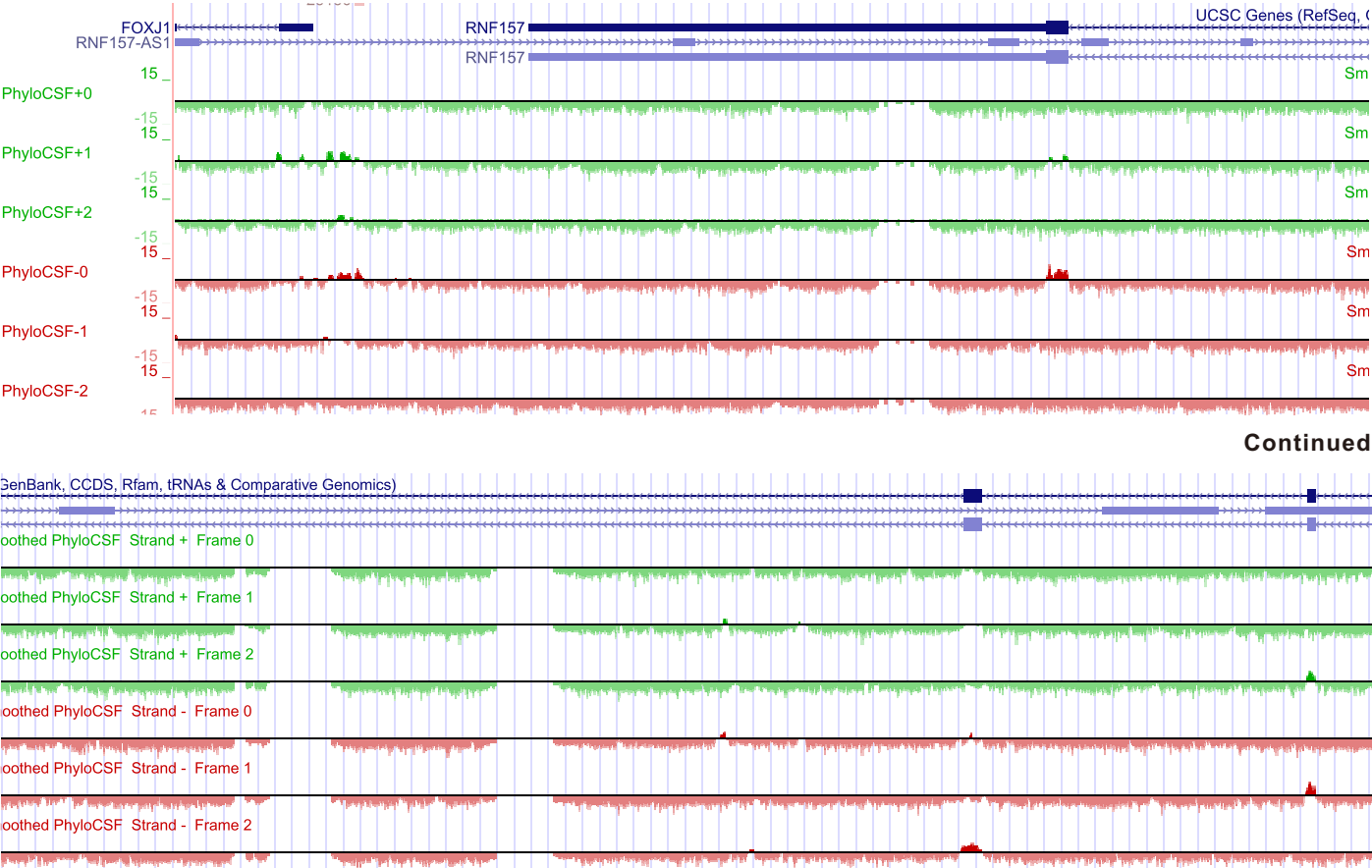

B

Open Reading Frame Viewer

Help

Sequence

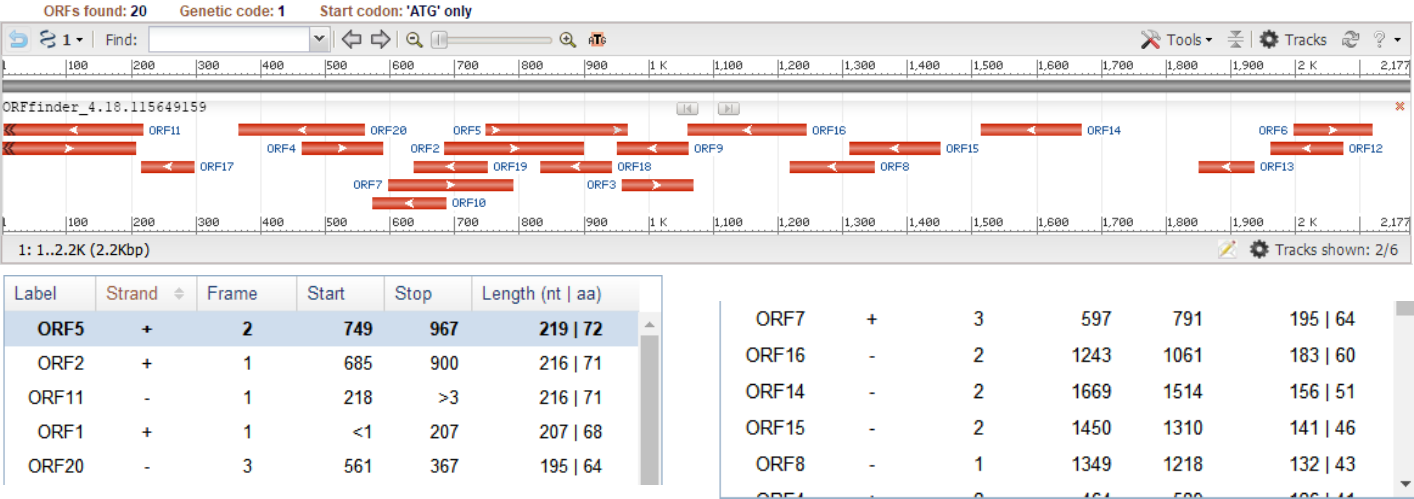

Continued

Supplement: Supplementary file 1 — Figure S1 [file 41419_2023_5668_MOESM1_ESM.pdf]

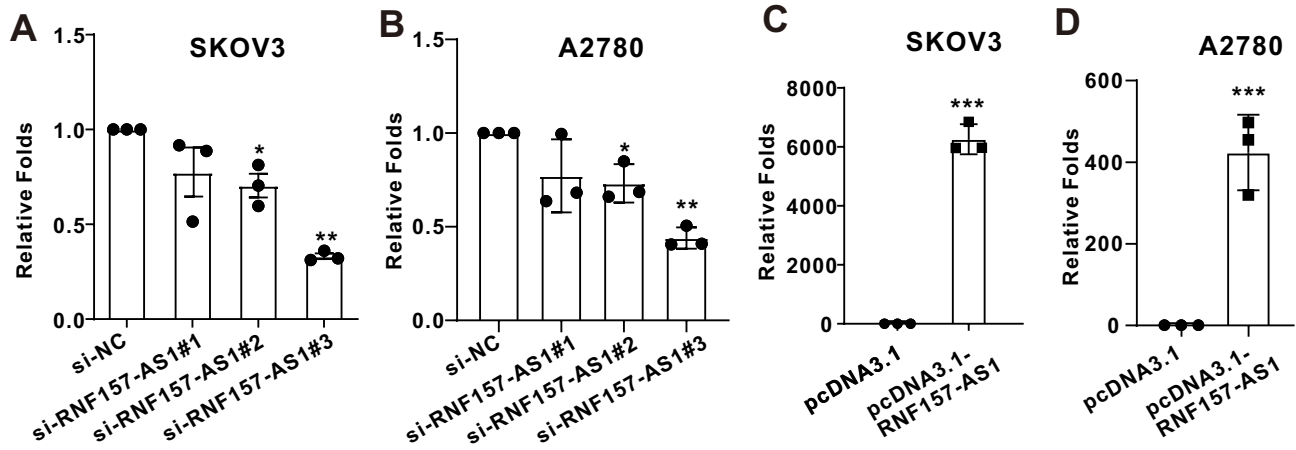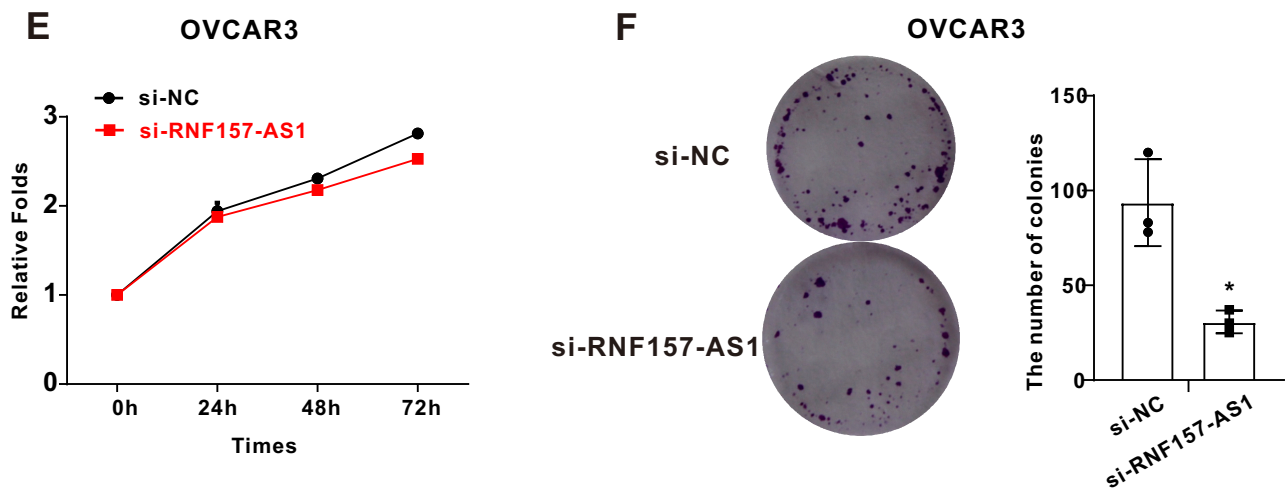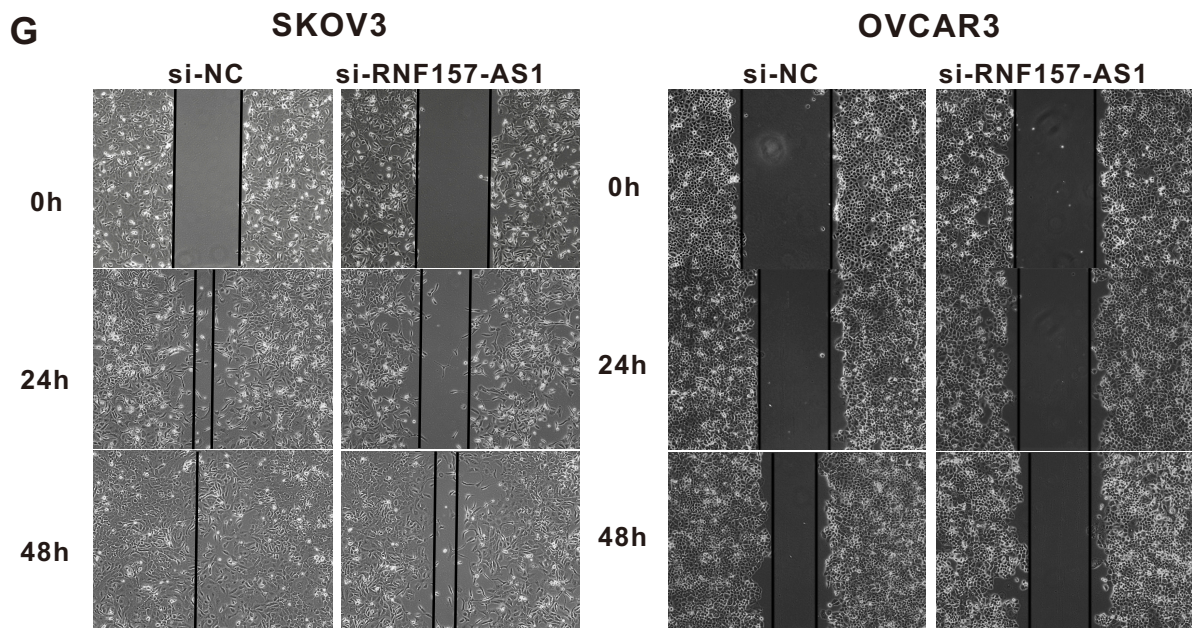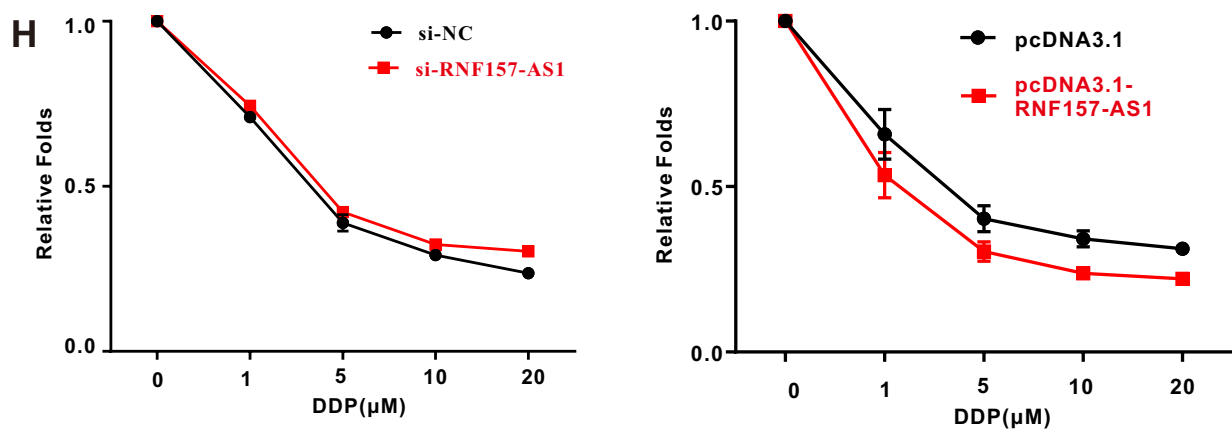

Supplement: Supplementary file 2 — Figure S2 [file 41419_2023_5668_MOESM2_ESM.pdf]

**A**

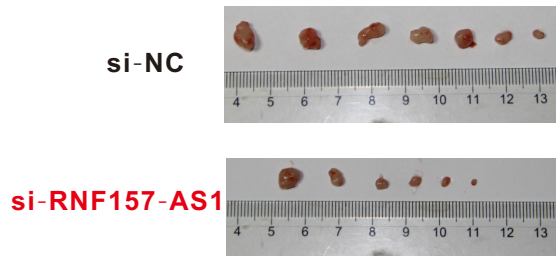

**B**

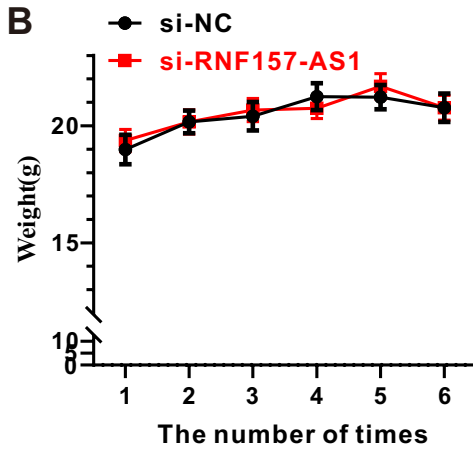

**C**

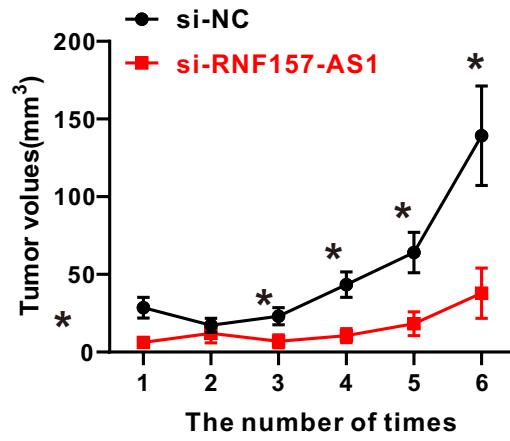

**D**

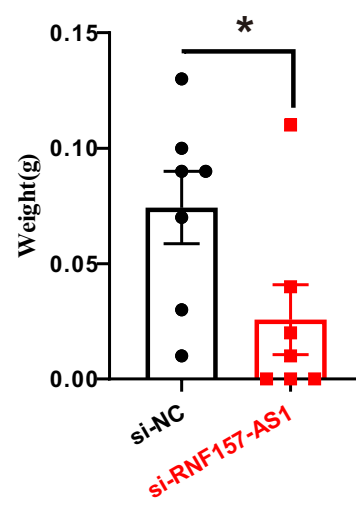

Supplement: Supplementary file 4 — Figure S4 [file 41419_2023_5668_MOESM4_ESM.pdf]

# SKOV3

**A**

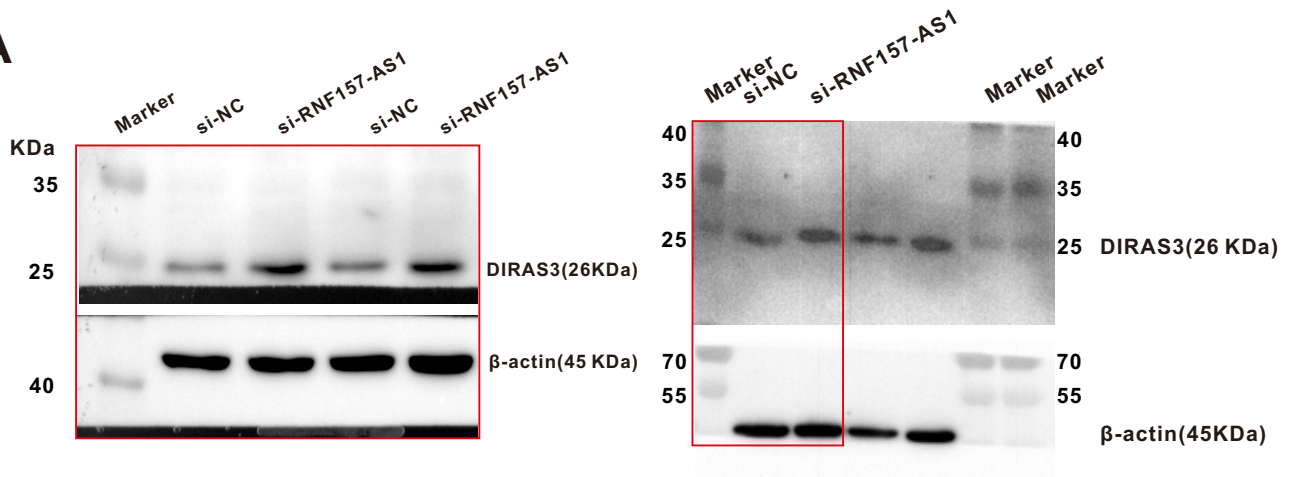

**B**

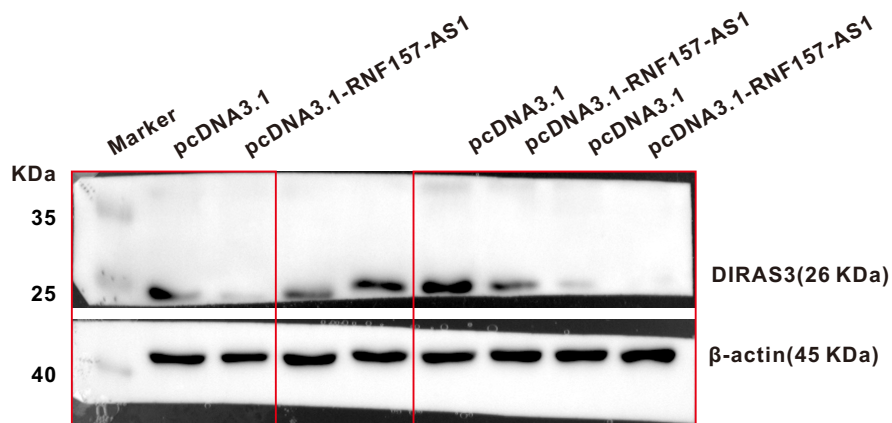

**C**

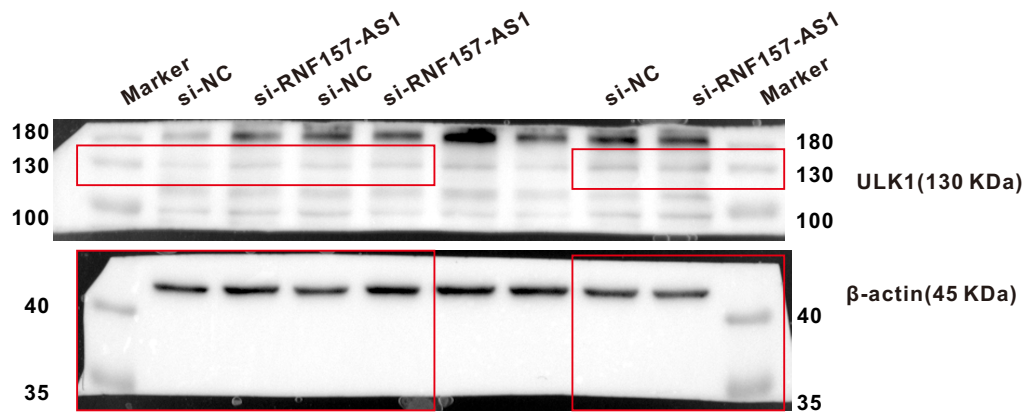

**D**

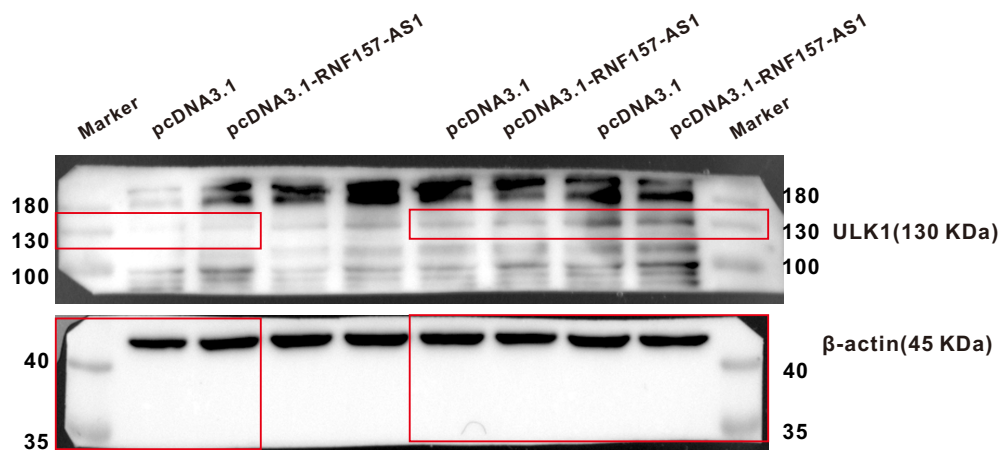

Supplement: Supplementary file 5 — Figure S5 [file 41419_2023_5668_MOESM5_ESM.pdf]

A2780

A

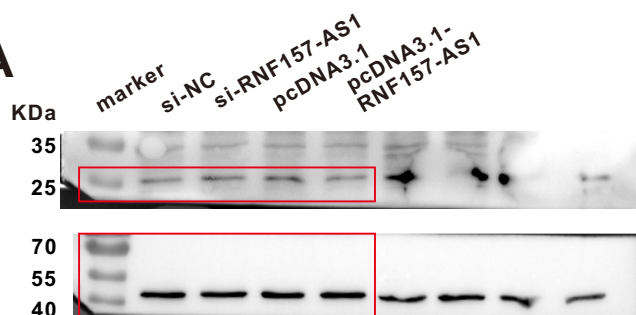

B

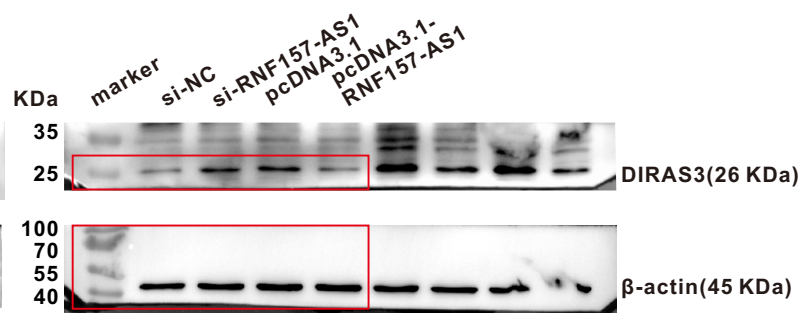

C

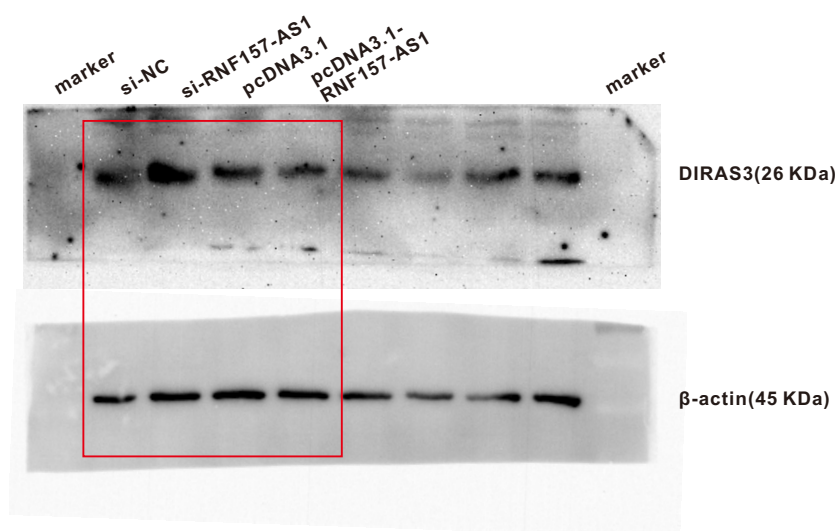

D

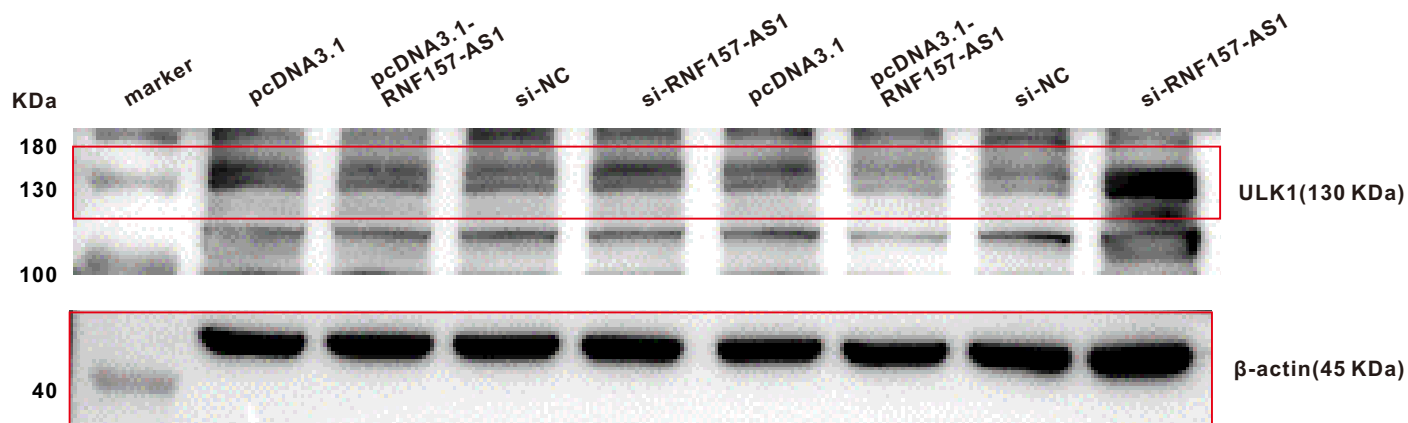

E

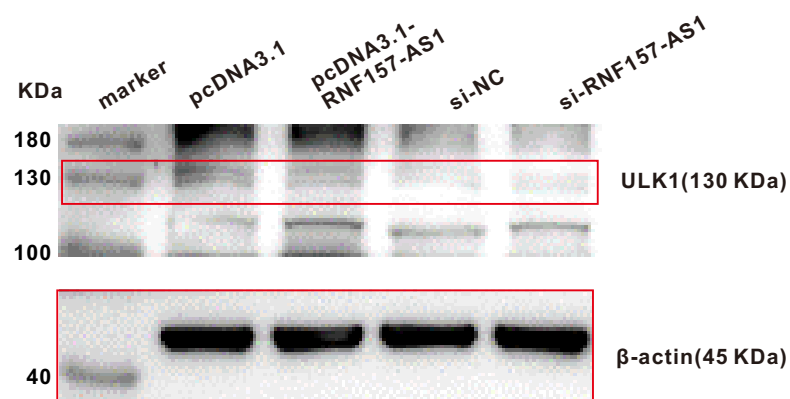

Supplement: Supplementary file 6 — Figure S6 [file 41419_2023_5668_MOESM6_ESM.pdf]

# SKOV3

**A**

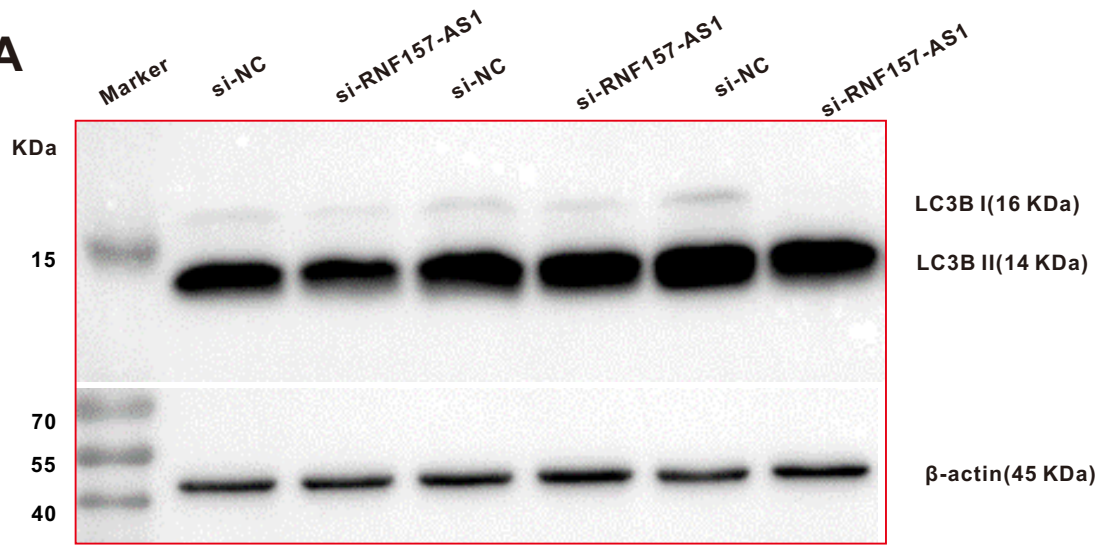

**B**

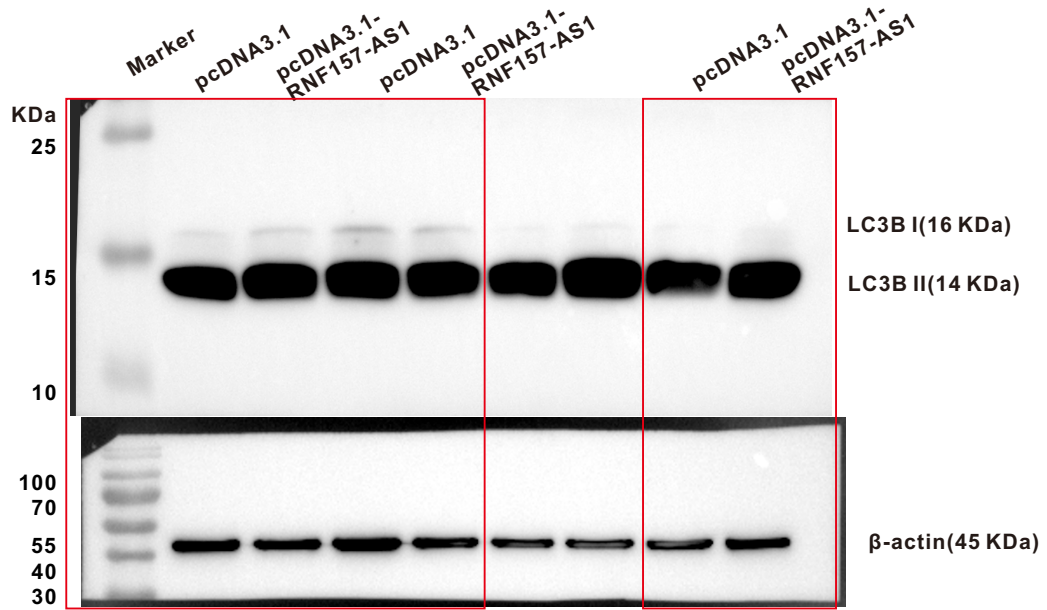

## A2780

**C**

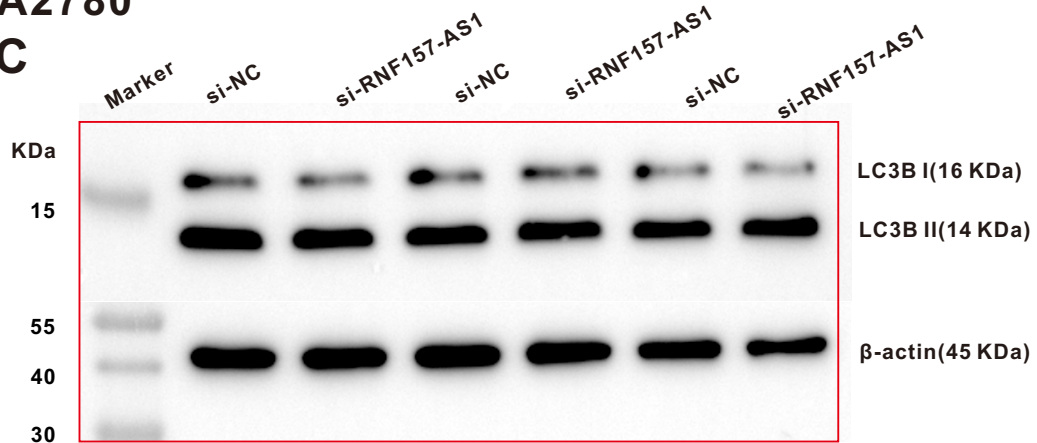

**D**

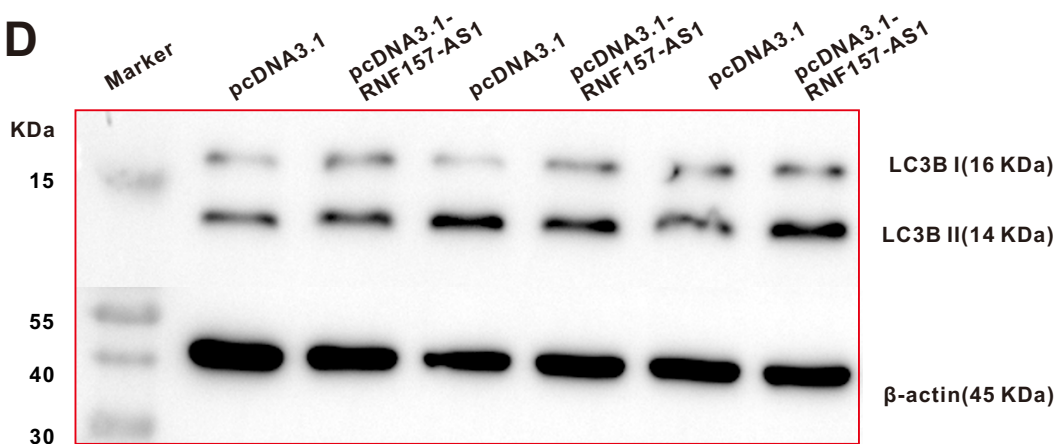

Supplement: Supplementary file 7 — Figure S7 [file 41419_2023_5668_MOESM7_ESM.pdf]

**A**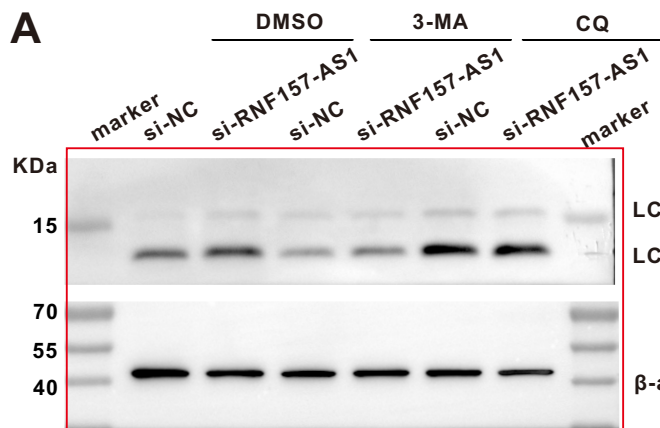**D**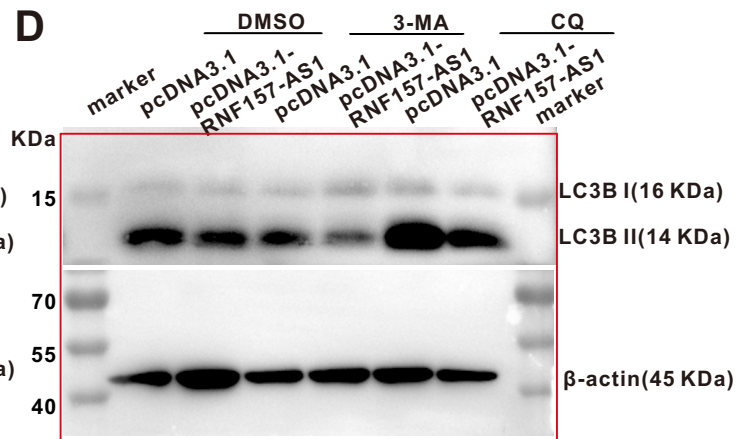**B**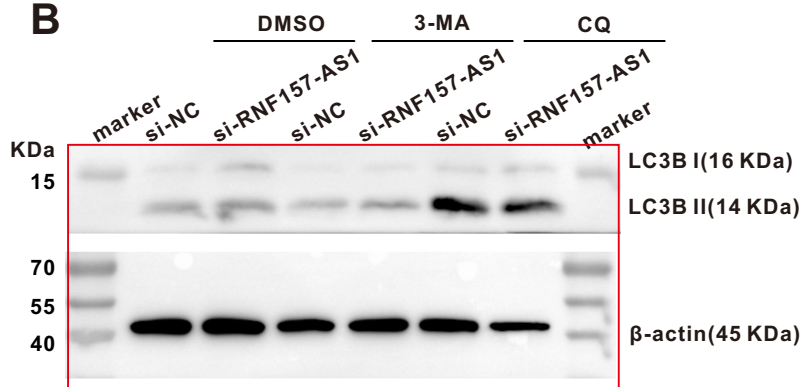**E**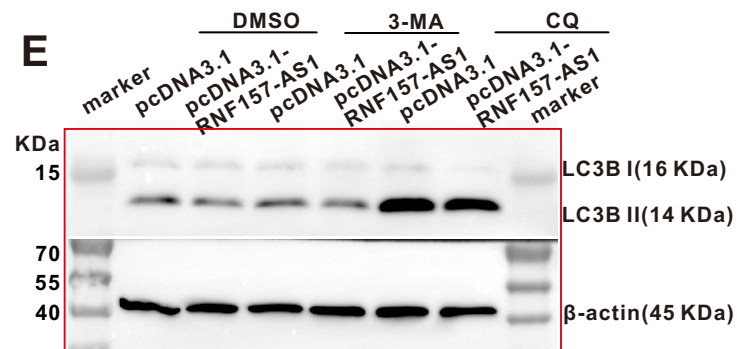**C**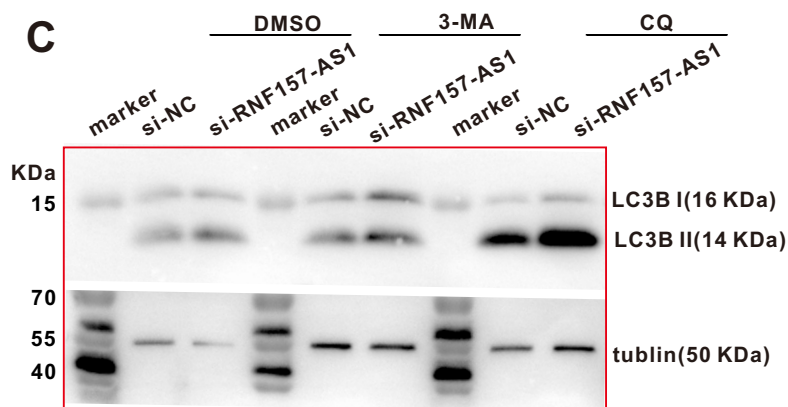**F**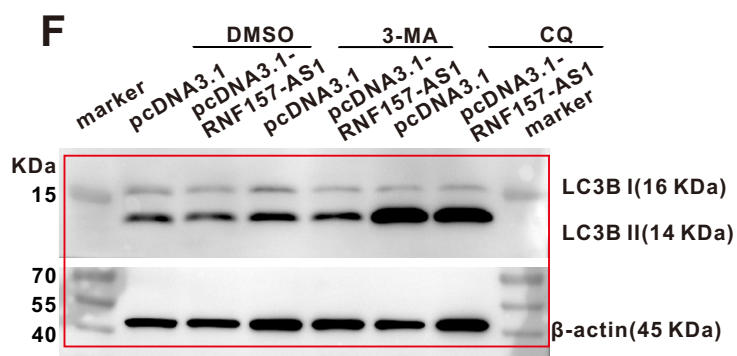

Supplement: Supplementary file 8 — Figure S8 [file 41419_2023_5668_MOESM8_ESM.pdf]
