# Supplementary material for: Differential effects of the LncRNA RNF157-AS1 on epithelial ovarian cancer cells through suppression of DIRAS3- and ULK1-mediated autophagy
Source: Cell Death Dis. 2023 Feb 20;14(2):140. doi: 10.1038/s41419-023-05668-5 (PMC9941098; doi:10.1038/s41419-023-05668-5)
Supplement: Supplementary file 9 — Table S1 [file 41419_2023_5668_MOESM9_ESM.docx]

**Table S1. The siRNA sequence of RNF157-AS1**

| **siRNA Name** | **Sequence (enter all sequence 5' to 3')** | **Special Codes** |
| --- | --- | --- |
| si-RNF157-AS1#1 | UGGAAACAACAUUCUUCCAACUGAA | S |
| si-RNF157-AS1#1 | UUCAGUUGGAAGAAUGUUGUUUCCA | AS |
| si-RNF157-AS1#2 | GGGCCUACGUUAAUAGCAUAUAUUU | S |
| si-RNF157-AS1#2 | AAAUAUAUGCUAUUAACGUAGGCCC | AS |
| si-RNF157-AS1#3 | ACUGUUUCUCUAAGGUGGAAGUAAA | S |
| si-RNF157-AS1#3 | UUUACUUCCACCUUAGAGAAACAGU | AS |
