# Supplementary material for: Differential effects of the LncRNA RNF157-AS1 on epithelial ovarian cancer cells through suppression of DIRAS3- and ULK1-mediated autophagy
Source: Cell Death Dis. 2023 Feb 20;14(2):140. doi: 10.1038/s41419-023-05668-5 (PMC9941098; doi:10.1038/s41419-023-05668-5)
Supplement: Supplementary file 10 — Table S2 [file 41419_2023_5668_MOESM10_ESM.docx]

**Table S2. The proteins were identified by LC-MS/MS in sense group**

| Protein FDR Confidence:  Combined | Accession | Gene Symbol | Abundance: F1:  Sample, antisense | Abundance: F2:  Sample, sense |
| --- | --- | --- | --- | --- |
| High | E9PKZ0 | RPL8 |  | 246228.05 |
| High | P17096 | HMGA1 |  | 226016.34 |
| High | P61009 | SPCS3 |  | 229514.3 |
| High | Q9UBQ7 | GRHPR |  | 211350.63 |
| High | E7EN89 | TOLLIP |  | 345812.66 |
| High | P49757 | NUMB |  | 240168.88 |
| High | P11766 | ADH5 |  | 159873.27 |
| High | J3QQM1 | PSMC5 |  | 501016.56 |
| High | A0A5F9ZHH9 | ACAT1 |  | 216644.63 |
| High | E9PNR6 | ARHGAP1 |  | 131242.17 |
| High | A0A2R8Y6J3 | RPL5 |  | 185605.03 |
| High | Q9H4A4 | RNPEP |  | 193128.5 |
| High | G3V4Q2 | SEC23A |  | 138743.23 |
| High | P08754 | GNAI3 |  | 175837.81 |
| High | B4DEH5 | LTA4H |  | 287398.78 |
| High | D6RAA6 | TMEM33 |  | 799611.44 |
| High | F8WBC0 | RAP1B |  | 1006719.6 |
