# Supplementary material for: Differential effects of the LncRNA RNF157-AS1 on epithelial ovarian cancer cells through suppression of DIRAS3- and ULK1-mediated autophagy
Source: Cell Death Dis. 2023 Feb 20;14(2):140. doi: 10.1038/s41419-023-05668-5 (PMC9941098; doi:10.1038/s41419-023-05668-5)
Supplement: Supplementary file 11 — Table S3 [file 41419_2023_5668_MOESM11_ESM.docx]

**Table S3. The qPCR primer sequences used in this work**

| **Gene symbol** | **Polarity** | **Sequence** |
| --- | --- | --- |
| RNF157-AS1^22^ | forward | 5'-GAGCCGTCAGGTCCACATG-3' |
|  | reverse | 5'-GTCATCCTATTGCTTCTGCTTTAGC-3' |
| DIRAS3^25^ | forward | 5'-TCTGCCCGCCCTGCTTAT-3' |
|  | reverse | 5'-TTGCCGTCGCCACTCTTG-3' |
| ULK1^28^ | forward | 5′-CAGACAGCCTGATGTGCAGT-3′ |
|  | reverse | 5′-CAGGGTGGGGATGGAGAT-3′ |
| 45S^37^ | forward | 5'-GTGCCCTCACGTGTTTCACTTT-3' |
|  | reverse | 5'-TAGGAGACAAACCTGGAACGCT-3' |
| 12S^37^ | forward | 5'-TCGATAAACCCCGCTCTACCT-3' |
|  | reverse | 5'-TGGCTACACCTTGACCTAACGTT -3' |
| β-actin | forward | 5'-AAGGCCAACCGCGAGAAG-3' |
|  | reverse | 5'-ACAGCCTGGATAGCAACGTACA-3' |
| DIRAS3-prom^25^ | forward | 5'-TCGATTGTTGTAGATGCCAAG-3' |
|  | reverse | 5'-AGACTTACCTTTCTCGGAGGC-3' |
| ULK1-prom^28^ | forward | 5′-TGCCCTGTTCCATATTTTGC-3′ |
|  | reverse | 5′-ACCCAAACCAACGACATAGC-3′ |
