# Supplementary material for: Differential effects of the LncRNA RNF157-AS1 on epithelial ovarian cancer cells through suppression of DIRAS3- and ULK1-mediated autophagy
Source: Cell Death Dis. 2023 Feb 20;14(2):140. doi: 10.1038/s41419-023-05668-5 (PMC9941098; doi:10.1038/s41419-023-05668-5)
Supplement: Supplementary file 12 — Supplemental figure legend [file 41419_2023_5668_MOESM12_ESM.docx]

**Figure S1.** **RNF157-AS1 is a long noncoding RNA. A.** According the UCSC Genome Browser, the value of phyloCSF in the exon of RNF157-AS1 was less than zero; **B.** According to PubMed ORFfinder, the largest ORF is 219 nt. Those results indicated that RNF157-AS1 is a long noncoding RNA.

**Figure S2. The effect of RNF157-AS1 on EOC cells. A&B**. Validation of siRNA interference of RNF157-AS1 in SKOV3 cells(A) and A2780 cells(B). The qPCR results showed that the interference efficiency of one siRNA(siRNA-3) more than 50%, then it was renamed as si-RNF157-AS1 in subsequent experiments; **C.** RNF157-AS1 overexpression efficiency was verified in SKOV3 cells; **D.** RNF157-AS1 overexpression efficiency was verified in a2780 cells; **E.** CCK8 assay suggested RNF157-AS1 depletion inhibited the proliferation of OVCAR3; **F.** Clone formation assay indicated that RNF157-AS1 knockdown significant inhibited the viability of OVCAR3. **G.** Wound healing assay indicated that RNF157-AS1 depletion decreased the migration of SKOV3and OVCAR3 cells; **H**. under DDP environment, RNF157-AS1 depletion increased OVCAR3 cells the viability while RNF157-AS1 overexpression decreased the viability of OVCAR3 cells.

**Figure S3. RNF157-AS1 serves as a modular scaffold to repress of DIRAS3 and ULK1 expression.** A. ChIP-qPCR assay followed by agarose gel electrophoresis was used to assess the binding of EZH2 to the promoter of DIRAS3 after knockdown or overexpressed RNF157-AS1; B. ChIP-qPCR assay followed by agarose gel electrophoresis was used to assess the binding of HMGA1 to the promoter of ULK1 after knockdown or overexpressed RNF157-AS1.

**Figure S4.** **Inhibition of tumor growth by siRNA-mediated knockdown of RNF157-AS1 in a xenograft mouse model. A.** SKOV3 tumors after removal from nude mice which is injected with si-NC and si-RNF157-AS1; **B.** The weights of nude mice were measured every week. The bars indicate SEM; **C.** The tumor volumes were calculated after injection every week; **D.** The tumors weights were calculated after removal from nude mice.

**Figure S5. Full-length blots of DIRAS3 and ULK1 in SKOV3 cell.**

**Figure S6. Full-length blots of DIRAS3 and ULK1 in A2780 cell.**

**Figure S7. Full-length blots of LC3 after RNF157-AS1 knockdown or overexpression in EOC cells.**

**Figure S8. Full-length blots of LC3 after the combination of RNF157-AS1 knockdown or overexpression and autophagy inhibitor in A2780 cell.**

**Table S1. The siRNA sequence of RNF157-AS1.**

**Table S2. The proteins were identified by LC-MS/MS in sense group.**

**Table S3. The qPCR primer sequences used in this work.**
