# Supplementary material for: Differential effects of the LncRNA RNF157-AS1 on epithelial ovarian cancer cells through suppression of DIRAS3- and ULK1-mediated autophagy
Source: Cell Death Dis. 2023 Feb 20;14(2):140. doi: 10.1038/s41419-023-05668-5 (PMC9941098; doi:10.1038/s41419-023-05668-5)
Supplement: Supplementary file 3 — Figure S3 [file 41419_2023_5668_MOESM3_ESM.pdf]

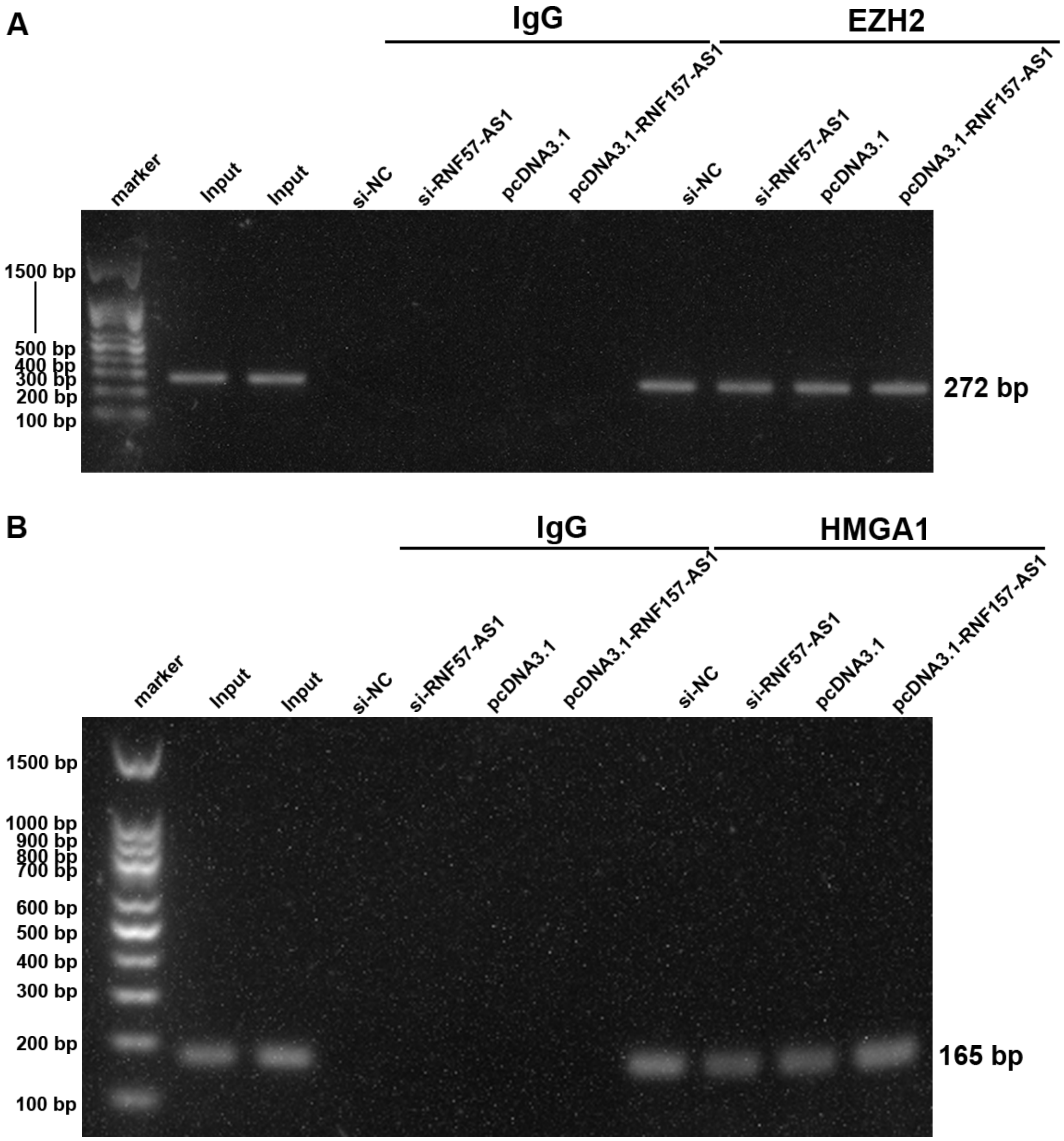
A
IgG
EZH2
marker
Input
Input
si-NC
si-RNF57-AS1
pcDNA3.1
pcDNA3.1-RNF157-AS1
si-NC
si-RNF57-AS1
pcDNA3.1
pcDNA3.1-RNF157-AS1
1500 bp
500 bp
400 bp
300 bp
200 bp
100 bp
272 bp
B
IgG
HMGA1
marker
Input
Input
si-NC
si-RNF57-AS1
pcDNA3.1
pcDNA3.1-RNF157-AS1
si-NC
si-RNF57-AS1
pcDNA3.1
pcDNA3.1-RNF157-AS1
1500 bp
1000 bp
900 bp
800 bp
700 bp
600 bp
500 bp
400 bp
300 bp
200 bp
100 bp
165 bp
